# Comparison of healthy lifestyle behaviors among individuals with and without cardiovascular diseases from urban and rural areas in China: A cross-sectional study

**DOI:** 10.1371/journal.pone.0181981

**Published:** 2017-08-03

**Authors:** Chuangshi Wang, Wei Li, Lu Yin, Jian Bo, Yaguang Peng, Yang Wang, on behalf of the PURE China Investigators

**Affiliations:** Medical Research and Biometrics Center, Fuwai Hospital, National Center for Cardiovascular Disease, Peking Union Medical College and Chinese Academy of Medical Sciences, Mentougou District, Beijing, China; Shanghai Diabetes Institute, CHINA

## Abstract

**Introduction:**

The study aimed to explore the gap of prevalence of healthy lifestyle behaviors including smoking cessation, quitting drinking, physical activity and healthy eating between Chinese adults with and without cardiovascular diseases (CVDs).

**Methods:**

This study is a cross-sectional component of Prospective Urban Rural Epidemiology (PURE)-China study, which recruited ~46,000 participants from 70 rural and 45 urban communities between 2005 and 2009. Participants were divided into disease (with CVDs) and control (without any diseases) groups. The adjusted rates were estimated for different strata by the generalized, linear mixed-effects model, including community as a random effect with additional adjustment for age, sex, education and income.

**Results:**

Among 40,490 participants, <10% had all four healthy lifestyle behaviors (disease group versus control group: urban areas: 7.8% versus 8.1%; rural areas: 3.4% versus 3.2%). The rates of smoking cessation and quitting drinking were significantly higher in disease group for both urban and rural residents (P<0.001). In urban areas, higher rates were observed in all other three healthy lifestyle behaviors except physical activity in low-income regions (P<0.05). Similarly, the higher trends were observed for stopping smoking and drinking while opposite trends for healthy eating among rural residents from low-income regions (P<0.05).

**Conclusions:**

Our study showed that the prevalence of adopting all four behaviors was low among Chinese adults. Individuals with CVDs were more likely to follow healthy lifestyle behaviors, but it still indicated a large gap between the actual and ideal adoption of healthy lifestyle behaviors, which called for the promotion of population-wide strategies to modify lifestyle behaviors in addition to individual health-care intervention strategies.

## Introduction

Cardiovascular diseases (CVDs) are the leading causes of death in the world as well as in China[1, 2]. In China, it is estimated that 290 million people have CVDs and about 3.5 million die annually from CVDs, accounting for more than 40% of all-cause deaths[3]. It is predicted that annual cardiovascular events increase by more than 50% between 2010 and 2030 in China based on population aging and growth alone [4].

A large percentage of CVDs, including coronary heart disease (CHD) and stroke, are caused by atherosclerosis. The main modifiable and behavioral risk factors for CVDs include tobacco usage, harmful usage of alcohol, physical inactivity and unhealthy diet which play an important role in the process of atherosclerosis by leading to physiological changes[1]. A large number of studies suggested that these factors were associated with higher risks of CVDs[5–10], while reduction of these unhealthy lifestyle behaviors was proved to be effective in the prevention of CVDs[11]. Additionally, adherence to behavioral modifications like exercise and smoking cessation was associated with lower risk of recurrent events or deaths after myocardial infarction (MI)[12].

Previous global studies reported that individuals after cardiovascular events showed poor and low healthy lifestyle behaviors [12, 13]. Several studies have examined the prevalence of individual lifestyle behavior among Chinese [14–19], but the prevalence of adopting four healthy lifestyle behaviors including smoking cessation, avoidance of alcohol consumption, high level of physical activity and healthy eating among adults with CVDs is still unknown. And it is still unclear about the gap of prevalence between individuals with and without CVDs. With the persistent increasing prevalence of CVDs in China[3], it is essential and important to examine the modifying behaviors with more details. Therefore, in this study we aimed to examine the prevalence of four major healthy lifestyle behaviors among Chinese adults with and without CVDs, respectively and explore their potential differences.

## Methods

Prospective Urban Rural Epidemiology (PURE) study[20] was a global cohort study which recruited 153,996 adults from 17 countries. One of the main objectives of the study was to examine the relationship between societal influences and the prevalence of risk factors and chronic non-communicable diseases measured at baseline, which could be considered the cross sectional component of study[20]. Its methods have been reported previously[20] and PURE-China study was part of it. In China, 46,285 adults were recruited from 12 provinces, which included 45 urban and 70 rural communities, at baseline between 2005 and 2009. This study came from the cross-sectional part of PURE-China study. Standardized approaches were employed for the whole recruitment process and for the data collection at each site. All site investigators were committed to collect high-quality data and tried to follow up the participants for 10 years or more. Households were eligible if at least one family member was between 35 to70 years old and the household intended to live at their current address for another 4 years. Written informed consent was obtained from all individual participants included in the study. This study was approved by Fuwai Hospital Ethics Committee.

### Study population

**Fig 1:**
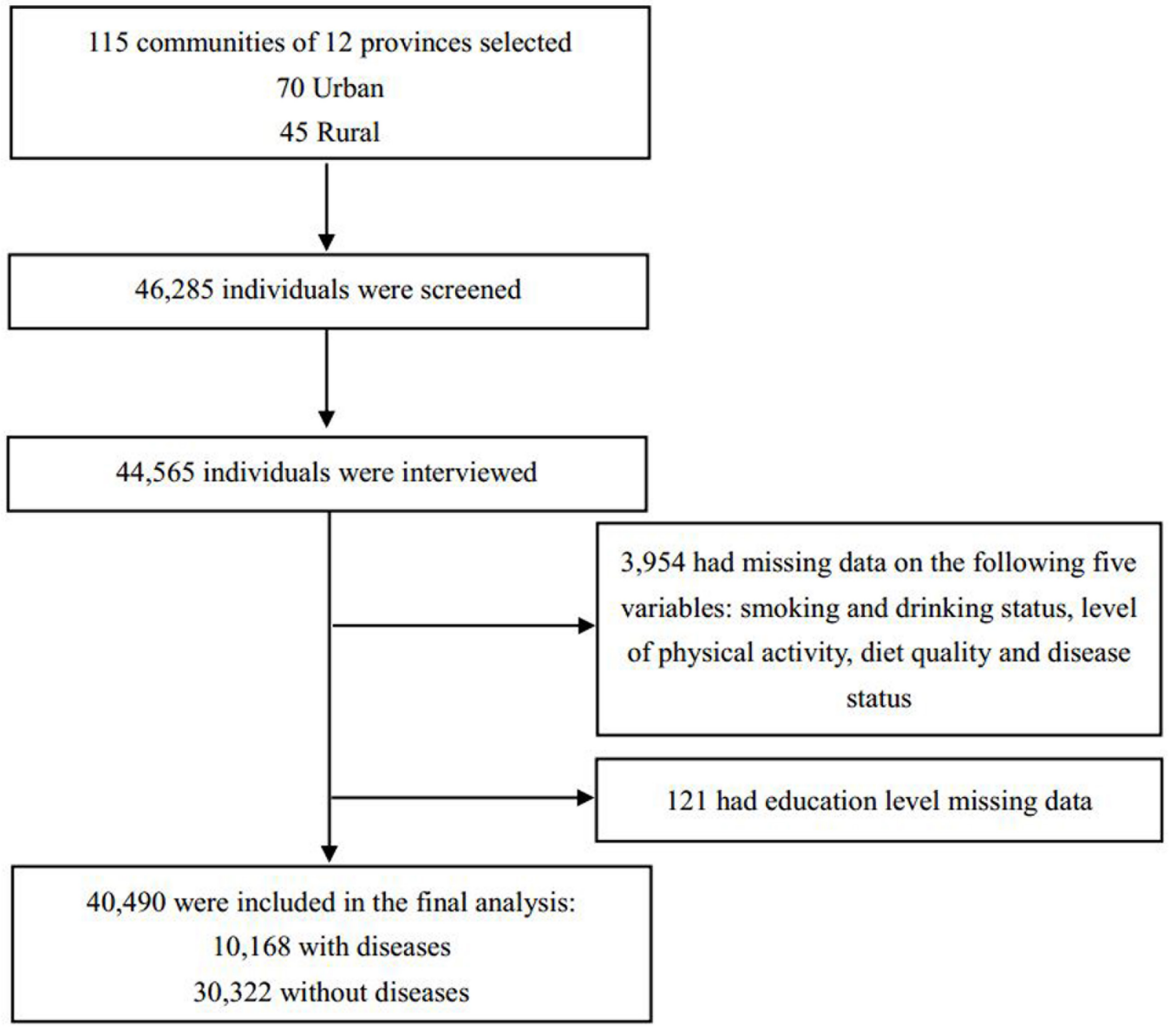
Participant enrollment and study population identification.

Of the 46,285 recruited, 44,565 individuals participated in all the three related sub-studies: adult study, physical activity questionnaire study and food frequency questionnaire study. After deleting 3,954 participants due to lack of data on profiles of lifestyle behaviors and disease conditions and 121 with missing data on education level, finally, a total of 40,490 individuals were included in the study: 10,168 had at least one CVD(disease group) and 30,322 were healthy (control group) [Fig 1].

### Definitions

#### Tobacco use

‘Former smokers’ are those who had quit smoking 1 year or more. Former smokers who had quit during the same year that their CVD events had occurred were deemed to have quit after the event. ‘Current smokers’ smoke at least 1 tobacco product daily for the last 12 months, including those individuals who have stopped smoking in the past year[21]. ‘Never smokers’ never used the tobacco products regularly. Smoking cessation rate is the proportion of current and former smokers who have stopped smoking.

#### Alcohol consumption

Self-reported alcohol use was recorded. Former drinking is defined as having stopped alcohol consumption for more than 1 year. Also, those who had quit drinking during the same year that their CVD events had occurred were deemed to stop alcohol consumption after the events. Current drinkers are individuals drinking alcohol in the previous year. Never drinking is defined as abstaining from alcoholic drink. Ever drinkers comprise the former and the current. Quitting drinking rate is the percentage of former ones among ever drinkers. In addition, based on the amount and frequency of alcohol consumption, we classified the drinkers into three groups: low (≤1 drink per week), moderate (1–7 drinks/week) and high intake (>7 drinks/week) by the tertiles.

#### Physical activity

International Physical Activity Questionnaire was used to gather information on job-, transport- and housework- related physical activity as well as during recreational, sports and leisure-time [22]. Participants were asked about the specific activities they did for 10 minutes or more during the past week by the intensity, the total duration per day and the total days of the week. We transformed the data into metabolic equivalent task (MET)-minutes per week and categorized the participants into 3 levels: low (≤600 MET-min/week), moderate (600–3000 MET-min/week) and high (≥3000 MET-min/week)[23].

#### Diet quality

Validated Food Frequency Questionnaire was applied to collect the data on food consumption and calculate the nutrients [24]and an adaptation of the Alternative Healthy Eating Index (AHEI), which is a highly predictive of cardiovascular disease risk[25, 26], was used to evaluate the diet quality for each participant. The scoring method on food quality has been described previously [27]. The scores of the tertiles with modified AHEI were used as the cut-off values for classification. Overall scores varied from 10.7 to 62.3, with higher scores suggesting higher quality. With the tertiles of 33.5 and 40.0, participants were classified as healthy, less healthy and unhealthy eating.

#### Cardiovascular diseases

Participants were asked about their history of diseases like CVDs including stroke and other diseases with standardized questionnaires. All diseases were based on self-report. CHD was defined as self-reported angina, MI, coronary artery bypass graft surgery, or percutaneous coronary intervention. As raised blood glucose or diabetes, one of the leading CVD risk factor, contributes to 6% of global deaths[1], people suffering from diabetes tend to change their lifestyle behaviors, therefore, individuals with CVDs or diabetes were allocated to the disease group. Participants without self-reporting of any diseases were deemed to be healthy and were considered as controls.

### Statistical analysis

Summary results of demographics were presented as counts (percentages) for categorical variables and means (standard deviation, SD) or medians (interquartile range, IQR) as appropriate for continuous variables. The prevalence of healthy lifestyle behaviors was summarized by proportions and 95% confidence intervals (CIs). According to the urban per capita annual disposable income and rural per capita net income in 2007 from National Bureau of Statistics of the People’s Republic of China, respectively, both urban and rural areas were divided into high- and low-income groups. Data analyses were performed by residence (urban and rural). Proportions were adjusted for age, sex, education and income as appropriate. In order to calculate the adjusted rates, generalized linear mixed-effects model was employed to take clustering effect into account (community as the random effect) with additional adjustments. Proportions and means were compared by chi-square test and Student T test or Kruskal-Wallis H test as appropriate. A 2-sided P < 0.05 was considered to be statistically significant. All statistical analyses were performed by SAS software (version 9.4, SAS Institute Inc., Cary, NC, USA) in Windows.

## Results

The process of participant enrollment in PURE-China study and the identification of study population were illustrated in Fig 1. Of the 40,490 participants, 20,095 were from urban areas and 20,395 from rural areas; 10,168 (25.1%) had a history of CVDs or stroke and 30,322 (74.9%) did not suffer from any surveyed diseases, which were deemed to be healthy. The mean age of the disease group was significantly greater than that of the control group (P<0.05). More participants with diseases lived in urban areas than those without diseases. Table 1 displayed the demographics of all the participants included in this study.

**Table 1:** Baseline characteristics of participants with and without cardiovascular diseases.

	No. of participants (%)	
	Disease group^a^ (n = 10,168)	Control group^a^ (n = 30,322)
Age, years		
Mean (SD)	56.5 (8.5)	49.4 (9.5)
Median (IQR)	57.1 (50.9–63.4)	49.1 (41.5–56.4)
Sex		
Female	6055 (59.6)	17718 (58.4)
Male	4113 (40.5)	12604 (41.6)
Education^b^		
Primary	3942 (38.8)	9481 (31.3)
Middle	5320 (52.3)	18173 (59.9)
High	906 (8.9)	2668 (8.8)
Type of community		
Urban	5993 (58.9)	14102 (46.5)
High-income	3190 (53.2)	7587 (53.8)
Low-income	2803 (46.8)	6515 (46.2)
Rural	4175 (41.1)	16220 (53.5)
High-income	3509 (84.1)	14632 (90.2)
Low-income	666 (16.0)	1588 (9.8)
Region		
East	5665 (55.7)	16317 (53.8)
Middle	2303 (22.7)	7022 (23.2)
West	2200 (21.6)	6983 (23.0)

Abbreviations: IQR, Interquartile Range; SD, Standard Deviation.

^a^Disease group, participants with cardiovascular diseases; Control group, participants without any diseases.

^b^High, trade school, college or university; Middle, secondary or high school; Low, primary or no education.

### Smoking

Overall, among ever smokers, significant difference of smoking cessation rate (P<0.0001) was observed between participants with disease [28.9% (95% CI, 27.2%-30.6%)] and without disease [13.3% (95% CI, 12.6%-14.1%)]. When analyzed by sex, more men (29.3% versus 13.2%, P<0.0001) and women (25.8% versus 15.0%, P<0.0001) stopped smoking in the disease group. Similar pattern was observed when analyzed by residence (S1 Table).

As shown in Table 2, significant number of participants from the disease group in urban areas stopped smoking after adjusting for age, sex, education and income (23.5% versus 16.6%, P<0.001). Similar trend was observed for individuals in high-income regions (P<0.001). In each group, the smoking cessation rates increased with income levels decreasing (P<0.05).

**Table 2:** Adjusted prevalence^a^ of smoking cessation, quitting drinking, high level of physical activity and healthy eating.

	Urban areas				Rural areas			
	Disease group^b^	Control group^b^	Overall	*p* values^c^	Disease group^b^	Control group^b^	Overall	*p* values^c^
**Smoking cessation**								
High-income	19.3 (14.9–24.7)	10.7 (8.1–14.0)	15.0 (11.9–18.7)	***<0.001	20.5 (16.3–25.5)	10.0 (7.9–12.6)	14.0 (11.2–17.5)	***<0.001
Low-income	28.0 (22.4–34.4)	24.4 (19.5–30.1)	25.7 (21.5–30.4)	0.08	55.6 (28.3–79.9)	37.1 (16.6–63.8)	46.9 (36.2–58.0)	*<0.05
Overall	23.5 (19.8–27.6)	16.6 (13.9–19.7)	-	***<0.001	36.4 (29.4–44.1)	20.1 (15.7–25.4)	-	***<0.001
*p* valves^c^	*<0.05	***<0.001	***<0.001		***<0.001	***<0.001	***<0.001	
**Quitting drinking**								
High-income	13.9 (9.7–19.4)	6.6 (4.6–9.4)	10.3 (7.4–14.0)	***<0.001	21.9 (16.4–28.6)	7.3 (5.3–10.0)	12.1 (8.8–16.3)	***<0.001
Low-income	31.0 (24.1–38.8)	13.3 (10.0–17.5)	20.7 (16.4–25.9)	***<0.001	38.4 (15.6–67.8)	18.8 (6.8–42.2)	39.5 (25.7–55.1)	**<0.01
Overall	21.9 (17.7–26.7)	9.6 (7.7–12.0)	-	***<0.001	35.8 (26.9–45.7)	13.8 (9.7–19.4)	-	***<0.001
*p* valves^c^	***<0.001	*<0.05	***<0.001		***<0.001	***<0.001	***<0.001	
**High level of physical activity**								
High-income	40.3 (30.9–50.6)	40.2 (30.9–50.4)	39.4 (31.0–48.3)	0.94	39.3 (33.2–45.9)	42.8 (36.6–49.2)	40.7 (34.9–46.9)	**<0.01
Low-income	41.1 (34.5–48.1)	42.5 (35.9–49.3)	42.7 (35.8–49.9)	0.29	37.2 (25.6–50.5)	45.1 (32.7–58.0)	43.4 (32.0–55.6)	**<0.01
Overall	40.8 (35.2–46.6)	41.3 (35.7–47.1)	-	0.55	40.0 (33.3–47.1)	44.1 (37.3–51.2)	-	***<0.001
*p* valves^c^	0.38	0.76	0.56		0.77	0.51	0.69	
**Healthy eating**								
High-income	26.4 (19.8–34.4)	27.9 (21.0–35.9)	26.8 (19.3–36.0)	0.17	27.1 (21.0–34.2)	25.3 (19.7–32.0)	27.2 (21.0–34.4)	0.06
Low-income	39.0 (30.6–48.0)	40.9 (32.5–49.9)	40.6 (32.7–48.9)	0.14	12.3 (5.2–26.2)	11.0 (4.7–23.5)	8.1 (4.1–15.3)	0.38
Overall	32.5 (26.7–38.8)	34.2 (28.3–40.6)	-	*<0.05	16.0 (11.2–22.2)	14.7 (10.4–20.6)	-	*<0.05
*p* valves^c^	*<0.05	*<0.05	*<0.05		***<0.001	***<0.001	***<0.001	
**Four healthy lifestyle behaviors**								
High-income	6.2 (4.2–8.9)	6.5 (4.5–9.2)	5.8 (3.9–8.7)	0.5	3.8 (2.5–5.6)	3.5 (2.3–5.1)	4.0 (2.7–5.7)	0.32
Low-income	10.0 (7.0–14.0)	10.4 (7.4–14.5)	10.7 (7.8–14.5)	0.45	3.9 (1.6–9.1)	3.7 (1.6–8.5)	2.7 (1.3–5.4)	0.79
Overall	7.8 (5.9–10.1)	8.1 (6.2–10.5)	-	0.34	3.4 (2.2–5.2)	3.2 (2.1–4.9)	-	0.52
*p* valves^c^	**<0.01	*<0.05	*<0.05		0.47	0.36	0.31	

^a^Adjusted for age, sex, education and income as appropriate based on mixed model.

^b^Disease group, participants with cardiovascular diseases; Control group, participants without any diseases.

^c^Boldface indicates statistical significance (*p<0.05, **p<0.01, ***p<0.001).

Similarly, for rural residents, higher smoking cessation rates in disease group were showed in both high- and low-income regions (P<0.05). And the pattern of increased smoking cessation rates with income decreasing was also observed in the two groups (P<0.001), as presented in Table 2.

### Alcohol consumption

About one-fourth (25.1%, 95% CI: 23.3%-26.8%) of individuals with diseases and less than 10% (8.8%, 95% CI: 8.2%-9.5%) in the control group had quit drinking alcoholic products which showed significant differences (P<0.0001). There were significant differences observed by sex, residence, education and income level in the two groups (P<0.0001 for all comparisons) (S1 Table).

In both urban and rural areas, the adjusted rates of stopping drinking were significantly higher among the participants with diseases regardless of their income levels (P<0.05). For each group, the quitting drinking rates showed an upward trend with the income level decreasing (P<0.05) (Table 2). Urban residents from low-income regions had a higher adjusted proportion of low alcohol intake compared with those from high-income regions (P<0.01). However, except urban participants from low-income regions, no significant difference was seen between disease group and control group after adjustment (S2 Table).

### Physical activity

The unadjusted proportions of participants undertaking high level of physical activity in the two groups were almost equal (approximately 43.5%). The adjusted rates of high level of physical activity were comparable between the two groups among urban residents. However, a significantly lower adjusted prevalence was observed in the disease group (P<0.05) for those living in rural areas, as presented in Table 2.

### Diet quality

Among individuals from urban communities, more healthy consumed a healthy diet (34.2% versus 32.5%, P<0.05). The trend that low-income regions had the higher prevalence of healthy diet intake was observed in both groups (P<0.05). In contrast, more participants with diseases in rural areas took a healthy diet (16.0% versus 14.7%, P<0.05). The opposite trend that high-income regions had the higher rate of healthy eating was observed in both the groups (P<0.001).

### Combination of healthy lifestyle behaviors

Overall, after adjustment, less than 10% of urban residents (disease group: 7.8%, 95% CI: 5.9%-10.1%; control group: 8.1%, 95% CI: 6.2%-10.5%) and not more than 5% of participants living in rural areas had all 4 healthy lifestyle behaviors (disease group: 3.4%, 95% CI: 2.2%-5.2%; control group: 3.2%, 95% CI: 2.1%-4.9%). Urban individuals from high-income regions had a lower prevalence of having all healthy lifestyle behaviors in both the groups (disease group: 6.2% versus 10.0%, P<0.01; control group: 6.5% versus 10.4%, P<0.05) (Table 2).

**Fig 2:**
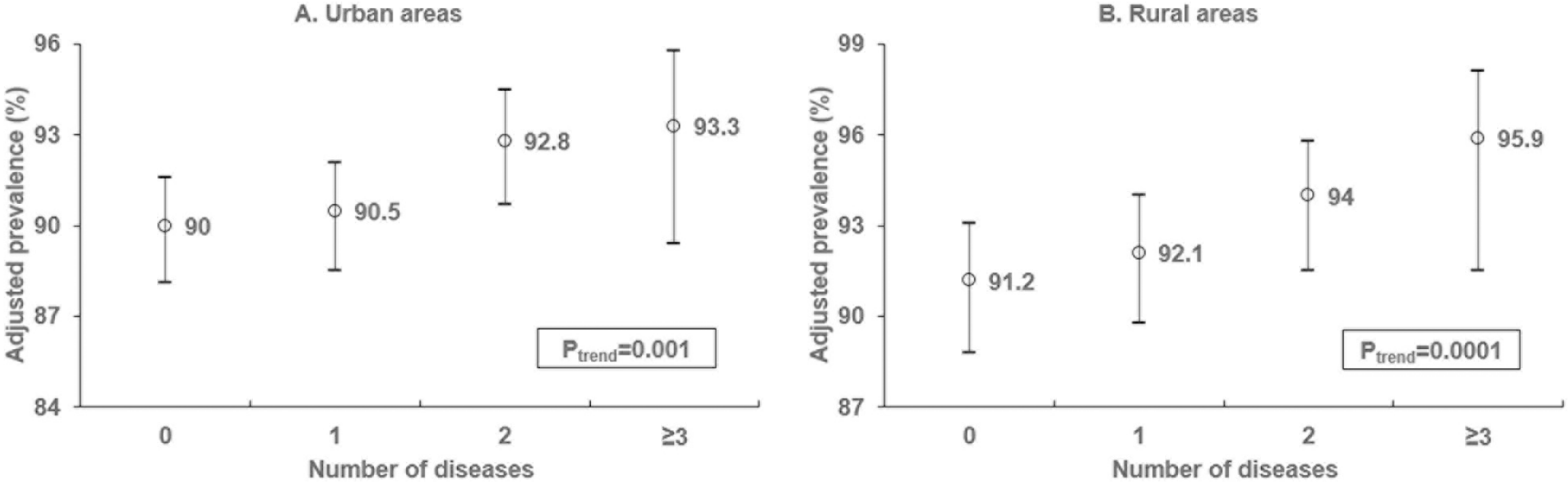
Adjusted prevalence of adoption of 2 or more healthy lifestyle behaviors by number of diseases.

Additionally, the adjusted prevalence of adopting ≥2 healthy lifestyle behaviors showed a graded increase with the number of CVDs that individuals suffering with growing for both urban and rural residents (P_trend_<0.01), as illustrated in Fig 2.

## Discussion

Our study indicated that there was a large gap that needed to bridge between the actual and expected adoption of the four healthy lifestyle behaviors which included avoidance of tobacco use and alcohol consumption, undertaking of high level of physical activity and healthy eating, although more individuals with CVDs tend to follow these behaviors than healthy ones. More than 70% of ever smokers with CVDs continued to smoke and only about 15% of the healthy stopped smoking. Around one-fourth and not more than one-tenth of ever drinkers quitted drinking in the disease and control groups. Less than half of the individuals undertook a high level of physical activity and only about one-third had a healthy diet in both the groups. Only one in ten had all four healthy lifestyle behaviors in the two groups. The results showed that the status of participation in healthy lifestyle behaviors for Chinese adults was grim and this calls for more attention to the primary prevention. Moreover, especially for individuals with CVDs from high-income regions, more focus should be given on strengthening the awareness and adoption of healthy lifestyle behaviors.

More individuals from high-income regions continued to smoke after cardiovascular events, which was likely to attribute to the affordability of health care. However, a previous study indicated an inverse relationship between the country income level and prevalence of tobacco use, which probably resulted from various approaches employed to control tobacco such as education on tobacco, smoking cessation programs, and active taxation policy as well as legislative measures in high-income countries as compared to the low-income countries[28]. One in 2 Chinese smokers were unaware that smoking causes heart disease and that the low smoking cessation rates are probably attributed to the poor awareness [29]. China is the world’s largest producer and consumer of tobacco products, and about 1 in every 3 cigarettes smoked in the world is smoked in China[30], which poses a big challenge. Of those men aged 40 to 74 years, smoking was estimated to cause nearly one-fourth of deaths due to CVDs[31]. There is little controversy over the beneficial effects of smoking cessation on CHD mortality. Therefore, in order to combat CVDs and improve the health of populations, comprehensive measures for tobacco control and promotion of smoking cessation need to be taken in China. In recent years, the Chinese government has taken severe actions for the prevention of tobacco use. For example, the tax on cigarettes has been increased from 5% to 11%, which has an efficient impact on reduction of cigarette purchases. Meanwhile, Beijing, the capital of China, became a smoke-free city, showing the government’s determination to create smoke-free environment and to combat CVDs. Additionally, other population-wide interventions like educational programs on smoking are the important components of efforts to improve the situation.

Overall, only a small proportion of individuals were observed who chose to stop consuming alcohol, although those with diseases had approximately a three-fold percentage of quitting drinking compared to the healthy. Like smoking cessation, individuals from high-income regions were less likely to stop drinking. The high-income regions, which could provide more convenient environments and resources like better hospitals, advanced medical facilities and experienced doctors for medical treatments, probably led to the poor situation that residents had less motivation to adopt healthy behaviors. In our study, as for the proportion of low alcohol intake, no significant difference was seen between disease group and control group except urban participants from low-income regions. The results of moderate alcohol consumption rates were similar. The relationship between the amount of alcohol consumption and CVDs is complicated. Light to moderate alcohol consumption might be associated with a reduced risk of multiple cardiovascular outcomes. However, heavy episodic drinking, which is defined as consuming 60 grams or more of pure alcohol on at least one occasion monthly, is thought to have hazardous effects[8, 32, 33]. The cut-off values for distinguishing heavy drinkers from light or moderate were inconsistent for different populations[34–36]. Overall, the disadvantages that alcohol consumption brings about outweigh its potential benefits at the population level[1]. Therefore, people tend to choose or be advised not to drink any more, especially after cardiovascular events occurred. And that probably be the reason why the difference of quitting drinking rates observed between disease group and control group was not seen in the proportion of low and moderate alcohol consumption. Till date in China, there were only a few policies and interventions existed to help control the alcohol consumption. More population-wide interventions that have greater impact should be implemented to prevent CVDs including national legal minimum age, time and place restrictions on sales of alcoholic beverages as well as legally required health warning labels on alcohol advertisements and containers. National government support and monitoring systems are also necessary. Moreover, further studies focusing on the dose-response relationships between alcohol consumption and CVDs in Chinese populations are encouraged.

The healthy had higher prevalence of high level of physical activity than the diseased in rural areas, which probably attributed to the certain degree of restriction of labor for work among the ill, whereas no difference was observed in the urban areas. As a whole, about two-fifths undertook high level of physical activity and only one-tenth took insufficient physical activity, which is defined as less than 5 times for 30 minutes of moderate activity or less than 3 times for 20 minutes of vigorous activity per week by World Health Organization or equivalent to 600 MET-min per week [1]. The activities related to job and housework occupied a great proportion with a low percentage of active activities in leisure time, which was consistent with the results reported in Du’s study[17]. The government should insist on encouraging exercise and generalize the mass sports activities by actively implementing the “National Fitness Program”.

Our study showed that about one-third of individuals had healthy diets. An inverse relationship between income level and the prevalence of high-quality diets was observed in urban areas. Nevertheless, rural individuals from high-income regions had a higher rate of healthy diets. This suggested that the income level might have different effects on the adoption of healthy diets in different areas. In our study, the poorest city had the higher income than the wealthiest country. As for those cities, high-fat and high-calorie foods like meat, fried foods and processed foods were more frequently consumed in the richer ones. While in rural areas, the wealthier ones were more likely to afford nutritious but healthy food such as milk, fresh fruits and vegetables. Given the numerous factors that affect the quality of diets like tastes, cooking preference, as well as availability and affordability of healthy foods, locally sensitive guidelines and scientific guidance for healthy diets should be developed in different regions. Moreover, with multi-sectoral cooperation and participation in China, interventions to improve public awareness regarding the importance of healthy diets and promote the consumption of healthy foods could be carried out effectively.

Cost effective and population-wide strategies to modify lifestyle behaviors as primary preventions across all populations are essential to prevent the CVDs, because some programs regarding lifestyle modifications were based on individual counseling which are unavailable to all the populations and are considered to be less effective. For individuals who have higher recurrent rates of CVDs, behavioral modifications as secondary preventions and individual health-care intervention strategies can go hand-in-hand to decline the recurrent event rates. Moreover, the poor public awareness of the cardiovascular benefits of healthy lifestyle behaviors calls for increased government investment in education programs to highlight its importance.

### Strengths and limitations

We collected data on lifestyle behaviors using standardized approaches from both urban and rural communities with varied income levels. This is the first study to compare the prevalence of healthy lifestyle behaviors between individuals with and without CVDs among Chinese adults in urban and rural areas. But, there were several limitations. Firstly, only baseline data were used to analyze in this study. For individuals who had CVDs, we were unable to determine the chronological order of events and adoption of these healthy lifestyle behaviors as the information obtained was only a snapshot of the lifestyle behaviors that the individuals had followed recently. Secondly, we identified the diseases based on self-reported data but did not verify these conditions with medical or hospital records. This might in turn lead to potential information bias related to diagnosis.

## Conclusions

Our study showed that the prevalence of adopting all four healthy lifestyle behaviors was low among individuals with and without CVDs among Chinese adults. Individuals with CVDs are more likely to follow healthy lifestyle behaviors, but it still indicated a large gap between the actual and ideal adoption of healthy lifestyle behaviors, which called for the promotion of population-wide strategies to modify lifestyle behaviors in addition to individual health-care intervention strategies. The variations in the relationships between lifestyle prevalence and income levels in different areas suggested locally sensitive but cost effective interventions were needed for the prevention and reduction of CVDs.

## Supporting information

S1 Table Prevalence of smoking cessation and quitting drinking among participants with and without cardiovascular diseases. (DOCX)

S2 Table Adjusted proportions of low and moderate intake among ever drinkers. (DOCX)

S1 File STROBE Statement—Checklist of items that should be included in reports of observational studies. (DOCX)

S2 File Study Questionnaire_EN. (PDF)

S3 File Study Questionnaire_CN. (PDF)

S4 File Data set. (SAS7BDAT)
