# Supplementary material for: Comparison of healthy lifestyle behaviors among individuals with and without cardiovascular diseases from urban and rural areas in China: A cross-sectional study
Source: PLoS One. 2017 Aug 3;12(8):e0181981. doi: 10.1371/journal.pone.0181981 (PMC5542534; doi:10.1371/journal.pone.0181981)
Supplement: S1 Table — (DOCX) [file pone.0181981.s001.docx]

**S1 Table: Prevalence of smoking cessation and quitting drinking among participants with and without cardiovascular diseases**

|  | **Disease Group**^a^ | | | **Control Group**^a^ | | |
| --- | --- | --- | --- | --- | --- | --- |
|  | **Overall** | **Women** | **Men** | **Overall** | **Women** | **Men** |
|  | **N(%, 95%CI)** | **N(%, 95%CI)** | **N(%, 95%CI)** | **N(%, 95%CI)** | **N(%, 95%CI)** | **N(%, 95%CI)** |
| **Smoking Cessation** |  |  |  |  |  |  |
| Participants | 808 (28.9, 27.2-30.6) | 73 (25.8, 20.8-31.3) | 735 (29.3, 27.5-31.1) | 1096 (13.3, 12.6-14.1) | 84 (15.0, 12.1-18.2) | 1012 (13.2, 12.5-14.0) |
| Location |  |  |  |  |  |  |
| Urban | 517 (31.8, 29.6-34.2) | 41 (27.2, 20.2-35.0) | 476 (32.3, 29.9-34.8) | 585 (17.4, 16.1-18.7) | 35 (17.2, 12.3-23.2) | 550 (17.4, 16.1-18.8) |
| High-income | 260 (30.1, 27.0-33.2) | 16 (21.6, 12.9-32.7) | 244 (30.9, 27.6-34.2) | 240 (12.6, 11.2-14.2) | 13 (10.6, 5.8-17.4) | 227 (12.8, 11.3-14.4) |
| Low-income | 257 (33.9,30.5-37.4) | 25 (32.5, 22.2-44.1) | 232 (34.0, 30.5-37.7) | 345 (23.5, 21.4-25.8) | 22 (27.5, 18.1-38.6) | 323 (23.3, 21.1-25.6) |
| Rural | 291 (24.9, 22.4-27.5) | 32 (24.2, 17.2-32.5) | 259 (25.0, 22.4-27.7) | 511 (10.5, 9.7-11.4) | 49 (13.7, 10.3-17.6) | 462 (10.3, 9.4-11.2) |
| High-income | 246 (22.7,20.2-25.3) | 30 (23.4, 16.4-31.7) | 216 (22.6, 20.0-25.4) | 423 (9.1, 8.3-10.0) | 44 (12.5, 9.2-16.4) | 379 (8.9, 8.0-9.8) |
| Low-income | 45 (52.9, 41.8-63.9) | 2 (50.0, 6.8-93.2) | 43 (53.1, 41.7-64.3) | 88 (38.4, 32.1-45.1) | 5 (71.4, 29.0-96.3) | 83 (37.4, 31.0-44.1) |
| Education |  |  |  |  |  |  |
| High | 105 (32.2, 27.2-37.6) | 2 (25.0, 3.2-65.1) | 103 (32.4, 27.3-37.5) | 149 (21.0, 18.1-24.2) | 3 (21.4, 4.7-50.8) | 146 (21.0, 18.0-24.2) |
| Middle | 440 (27.2, 25.1-29.5) | 26 (26.3, 17.9-36.1) | 414 (27.3, 25.1-29.6) | 634 (12.0, 11.1-12.9) | 36 (13.3, 9.5-18.0) | 598 (11.9, 11.0-12.8) |
| Low | 263 (30.9, 27.8-34.1) | 45 (25.6, 19.3-32.7) | 218 (32.3, 28.8-36.0) | 313 (14.2, 12.8-15.7) | 45 (16.2, 12.1-21.1) | 268 (13.9, 12.4-15.5) |
| **Quitting Drinking** |  |  |  |  |  |  |
| Participants | 599 (25.1,23.3-26.8) | 82 (29.2, 23.9-34.9) | 517 (24.5, 22.7-26.4) | 659 (8.8, 8.2-9.5) | 126 (12.4, 10.4-14.6) | 533 (8.3, 7.6-9.0) |
| Location |  |  |  |  |  |  |
| Urban | 347 (25.8, 23.5-28.3) | 54 (30.0, 23.4-37.3) | 293 (25.2, 22.7-27.8) | 332 (10.1, 9.1-11.2) | 79 (13.0, 10.4-15.9) | 253 (9.4, 8.4-10.6) |
| High-income | 128 (19.1, 16.2-22.3) | 14 (18.4, 10.5-29.0) | 114 (19.2, 16.1-22.6) | 122 (7.5, 6.3-8.9) | 16 (8.9, 5.2-14.1) | 106 (7.4, 6.1-8.8) |
| Low-income | 219 (32.6, 29.1-36.3) | 40 (38.5, 29.1-48.5) | 179 (31.5, 27.7-35.5) | 210 (12.6, 11.0-14.3) | 63 (14.6, 11.4-18.3) | 147 (11.9, 10.1-13.8) |
| Rural | 252 (24.1, 21.5-26.8) | 28 (27.7, 19.3-37.5) | 224 (23.7, 21.0-26.5) | 327 (7.8, 7.0-8.7) | 47 (11.5, 8.6-15.0) | 280 (7.4, 6.6-8.3) |
| High-income | 206 (21.7, 19.1-24.5) | 26 (28.3, 19.1-37.5) | 180 (21.0, 18.3-23.9) | 261 (6.7, 6.0-7.6) | 40 (10.9, 7.9-14.5) | 221 (6.3, 5.5-7.1) |
| Low-income | 46 (46.0, 36.0-56.3) | 2 (22.2, 2.8-60.0) | 44 (48.4, 37.7-59.1) | 66 (22.6, 17.9-27.8) | 7 (17.5, 7.3-32.8) | 59 (23.4, 18.3-29.1) |
| Education |  |  |  |  |  |  |
| High | 82 (24.3, 19.8-29.2) | 8 (23.5, 10.8-41.2) | 74 (24.3, 19.6-29.6) | 97 (9.8, 8.0-11.8) | 23 (11.8, 7.6-17.2) | 74 (9.3, 7.4-11.5) |
| Middle | 310 (22.9, 20.7-25.2) | 33 (25.2, 18.0-33.5) | 277 (22.6, 20.3-25.1) | 361 (7.9, 7.1-8.7) | 55 (10.4, 7.9-13.3) | 306 (7.6, 6.8-8.4) |
| Low | 207 (29.7, 26.3-33.2) | 41 (35.3, 26.7-44.8) | 166 (28.5, 24.9-32.4) | 201 (10.5, 9.2-12.0) | 48 (16.3, 12.3-21.1) | 153 (9.5, 8.1-11.0) |

Abbreviation: CI, Confidence Interval.

^a^Disease group, participants with cardiovascular diseases; Control group, participants without any diseases.
