# Supplementary material for: Comparison of healthy lifestyle behaviors among individuals with and without cardiovascular diseases from urban and rural areas in China: A cross-sectional study
Source: PLoS One. 2017 Aug 3;12(8):e0181981. doi: 10.1371/journal.pone.0181981 (PMC5542534; doi:10.1371/journal.pone.0181981)
Supplement: S2 Table — (DOCX) [file pone.0181981.s002.docx]

**S2 Table: Adjusted proportions**^a^ **of low and moderate intake among ever drinkers**

|  | **Disease group**^b^ | **Control group**^b^ | **Overall** | ***p* values** |
| --- | --- | --- | --- | --- |
| **Proportion of low intake** | |  |  |  |
| **Urban areas** | | | | |
| High-income | 38.9 (31.6-46.7) | 37.5 (31.0-44.5) | 39.0 (33.6-44.7) | 0.59 |
| Low-income | 47.3 (41.3-53.4) | 55.2 (50.3-59.9) | 50.3 (45.3-55.2) | **^**^<0.01** |
| Overall | 42.8 (38.2-47.7) | 46.4 (42.3-50.5) | - | 0.07 |
| *p* valves | 0.21 | **^**^<0.01** | **^**^<0.01** |  |
| **Rural areas** |  |  |  |  |
| High-income | 39.0 (31.1-47.4) | 39.0 (31.8-46.7) | 38.4 (31.5-45.7) | 0.99 |
| Low-income | 42.4 (25.2-61.7) | 38.0 (23.8-54.5) | 51.1 (36.7-65.4) | 0.49 |
| Overall | 44.9 (35.9-54.3) | 44.4 (35.9-53.2) | - | 0.80 |
| *p* valves | **^**^<0.01** | 0.12 | 0.10 |  |
| **Proportion of moderate intake** | |  |  |  |
| **Urban areas** |  |  |  |  |
| High-income | 43.0 (36.3-49.9) | 44.5 (38.5-50.6) | 42.5 (38.0-47.1) | 0.55 |
| Low-income | 38.6 (33.6-43.8) | 34.8 (31.1-38.6) | 38.0 (34.2-41.9) | 0.11 |
| Overall | 40.8 (36.8-45.0) | 39.6 (36.3-43.0) | - | 0.49 |
| *p* valves | 0.47 | 0.07 | 0.09 |  |
| **Rural areas** |  |  |  |  |
| High-income | 41.0 (34.8-47.4) | 45.4 (39.8-51.3) | 43.8 (38.3-49.3) | **^*^<0.05** |
| Low-income | 46.9 (30.1-64.4) | 53.3 (38.6-67.3) | 43.0 (33.2-53.3) | 0.34 |
| Overall | 41.1 (34.5-48.0) | 45.7 (39.3-52.2) | - | **^*^<0.05** |
| *p* valves | 0.37 | 0.93 | 0.88 |  |

^a^Adjusted for age, sex, education and income as appropriate based on mixed model.

^b^Disease group, participants with cardiovascular diseases; Control group, participants without any diseases.

^c^Boldface indicates statistical significance (^*^*p*<0.05, ^**^*p*<0.01, ^***^*p*<0.001)
