# Supplementary material for: Comparison of healthy lifestyle behaviors among individuals with and without cardiovascular diseases from urban and rural areas in China: A cross-sectional study
Source: PLoS One. 2017 Aug 3;12(8):e0181981. doi: 10.1371/journal.pone.0181981 (PMC5542534; doi:10.1371/journal.pone.0181981)
Supplement: S1 File — (DOCX) [file pone.0181981.s003.docx]

**STROBE Statement—checklist of items that should be included in reports of observational studies**

|  | Item No. | Recommendation | Page  No. | Relevant text from manuscript |
| --- | --- | --- | --- | --- |
| **Title and abstract** | 1 | (*a*) Indicate the study’s design with a commonly used term in the title or the abstract | 1, 2 | Cross-sectional study/component |
|  |  | (*b*) Provide in the abstract an informative and balanced summary of what was done and what was found | 2 | Methods and Results |
| Introduction | | | |  |
| Background/rationale | 2 | Explain the scientific background and rationale for the investigation being reported | 3 | Prevention of CVDs, healthy lifestyle behaviors |
| Objectives | 3 | State specific objectives, including any prespecified hypotheses | 3 | Examine the prevalence, explore potential differences |
| Methods | | | |  |
| Study design | 4 | Present key elements of study design early in the paper | 4 | Prospective, cohort study, cross-sectional part |
| Setting | 5 | Describe the setting, locations, and relevant dates, including periods of recruitment, exposure, follow-up, and data collection | 4 | 45 urban and 70 rural communities, from 12 provinces, between 2005 and 2009, follow up for 10 years or more |
| Participants | 6 | (*a*) *Cohort study*—Give the eligibility criteria, and the sources and methods of selection of participants. Describe methods of follow-up  *Case-control study*—Give the eligibility criteria, and the sources and methods of case ascertainment and control selection. Give the rationale for the choice of cases and controls  *Cross-sectional study*—Give the eligibility criteria, and the sources and methods of selection of participants | 4 | At least one family member was between 35 to 70 years old, live at their current address for another 4 years, written informed consent |
|  |  | (*b*) *Cohort study*—For matched studies, give matching criteria and number of exposed and unexposed  *Case-control study*—For matched studies, give matching criteria and the number of controls per case | NA | NA |
| Variables | 7 | Clearly define all outcomes, exposures, predictors, potential confounders, and effect modifiers. Give diagnostic criteria, if applicable | 5-6 | Definitions, tobacco use, alcohol consumption, physical activity, diet, CVDs |
| Data sources/ measurement | 8* | For each variable of interest, give sources of data and details of methods of assessment (measurement). Describe comparability of assessment methods if there is more than one group | *5-6* | *Questionnaires, the specific activities they did for 10 minutes or more, MET* |
| Bias | 9 | Describe any efforts to address potential sources of bias | NA | NA |
| Study size | 10 | Explain how the study size was arrived at | NA | NA |
| Quantitative variables | 11 | Explain how quantitative variables were handled in the analyses. If applicable, describe which groupings were chosen and why | 5-6 | MET, AHEI, tertiles, scoring method has been described elsewhere |
| Statistical methods | 12 | (*a*) Describe all statistical methods, including those used to control for confounding | 7 | Generalized linear mixed model, random effect |
|  |  | (*b*) Describe any methods used to examine subgroups and interactions | NA | NA |
|  |  | (*c*) Explain how missing data were addressed | 4 | Delete 121 with missing education level |
|  |  | (*d*) *Cohort study*—If applicable, explain how loss to follow-up was addressed  *Case-control study*—If applicable, explain how matching of cases and controls was addressed  *Cross-sectional study*—If applicable, describe analytical methods taking account of sampling strategy | 7 | take clustering into effect |
|  |  | (*e*) Describe any sensitivity analyses | NA | NA |
| Results | | | | |
| Participants | 13* | (a) Report numbers of individuals at each stage of study—eg numbers potentially eligible, examined for eligibility, confirmed eligible, included in the study, completing follow-up, and analysed | *7, Fig 1* | *Identification of study population* |
|  |  | (b) Give reasons for non-participation at each stage | *4, Fig 1* | *Lack of profiles* |
|  |  | (c) Consider use of a flow diagram | *Fig 1* | *Fig 1* |
| Descriptive data | 14* | (a) Give characteristics of study participants (eg demographic, clinical, social) and information on exposures and potential confounders | *7-8, table 1* | *Baseline characteristics of participants* |
|  |  | (b) Indicate number of participants with missing data for each variable of interest | *NO* | *NA* |
|  |  | (c) *Cohort study*—Summarise follow-up time (eg, average and total amount) | *NA* | *NA* |
| Outcome data | 15* | *Cohort study*—Report numbers of outcome events or summary measures over time | *NA* | *NA* |
|  |  | *Case-control study—*Report numbers in each exposure category, or summary measures of exposure | *NA* | *NA* |
|  |  | *Cross-sectional study—*Report numbers of outcome events or summary measures | *S1 Table* | *Number of quitting smoking* |
| Main results | 16 | (*a*) Give unadjusted estimates and, if applicable, confounder-adjusted estimates and their precision (eg, 95% confidence interval). Make clear which confounders were adjusted for and why they were included | 8-12,table 2 and S1 Table | Adjusted prevalence, 95% CI |
|  |  | (*b*) Report category boundaries when continuous variables were categorized | 6 | tertiles |
|  |  | (*c*) If relevant, consider translating estimates of relative risk into absolute risk for a meaningful time period | NA | NA |
| Other analyses | 17 | Report other analyses done—eg analyses of subgroups and interactions, and sensitivity analyses | NA | NA |
| Discussion | | | | |
| Key results | 18 | Summarise key results with reference to study objectives | 12-13 | Large gap, more individuals with CVDs tended to follow healthy lifestyle behaviors, only one in ten had all four healthy lifestyle behaviors |
| Limitations | 19 | Discuss limitations of the study, taking into account sources of potential bias or imprecision. Discuss both direction and magnitude of any potential bias | 16 | Self-report, potential information bias |
| Interpretation | 20 | Give a cautious overall interpretation of results considering objectives, limitations, multiplicity of analyses, results from similar studies, and other relevant evidence | 15-16 | Costly effective and population-wide strategies, essential, highlight its importance |
| Generalisability | 21 | Discuss the generalisability (external validity) of the study results | NA | NA |
| Other information | |  | | |
| Funding | 22 | Give the source of funding and the role of the funders for the present study and, if applicable, for the original study on which the present article is based | NA | Not in the manuscript but provide the information when submission |

*Give information separately for cases and controls in case-control studies and, if applicable, for exposed and unexposed groups in cohort and cross-sectional studies.
