# Supplementary material for: Comparison of healthy lifestyle behaviors among individuals with and without cardiovascular diseases from urban and rural areas in China: A cross-sectional study
Source: PLoS One. 2017 Aug 3;12(8):e0181981. doi: 10.1371/journal.pone.0181981 (PMC5542534; doi:10.1371/journal.pone.0181981)
Supplement: S2 File — (PDF) [file pone.0181981.s004.pdf]

PURE/International

We are very grateful to you for your participation in this study. All information given by you will be held in strict confidence, and will be used for the purpose of this study only after removing any personal identifying information.

Adult Questionnaire

INSTRUCTIONS

Please answer EACH question by marking an X in ONE BOX on each line: (unless otherwise instructed)

[X]

OR

By writing number(s) in the spaces provided:

[1] [8]

OR

By specifying the answer on the line(s) provided

October 2012

Adult Questionnaire

Subject Initials- F= first letter of first name
M= first letter of middle name
L= first letter of last name

India: Remove ID #’s from all pages except the first and the last.

Exclude question #2 for all countries except India

3. National I.D#

If not applicable please mark the N/A box

Exclude question 3a) for all countries except Canada

6. Marital Status:

UAE: Exclude → Common law/Living with partner, Seperated

IRAN: Exclude → Common law/Living with partner

Ethnicity Codes

01 **-** South Asian (India, Sri Lanka, Pakistan, Bangladesh)

02 **-** Chinese (China, Hong Kong, Taiwan)

03 - Japanese

04 - Malays

05 **-** Other Asian (Korea, Malaysia, Papua New Guinea, Thailand, Philippines, Indonesia, Nepal,Vietnam, Cambodia, Laos, Myanmar/Burma, Bhutan, Singapore)

06 **-** Persian

07 **-** Arab

08 **-** Black African

09 **-** Coloured African (Subsaharan African only)

10 **-** European

11 **-** Native North/South American or Australian Aborigine

12 **-** Latin American (Latino)

13 **-** Bantu/Semi Bantu

14 **-** Hemitic/Semi Hemitic

15 **-** Nilotic/Hausa

16 **-** Pygmie

17 **-** Swahili

18 **-** Other (any other ethnoracial group not listed above)

Q7 → Exclude in Poland

Q8 → Exclude in all countries except India, Kenya, South Africa and Zimbabwe
Ghana - Exclude Caste; keep Tribe

Q9 → Exclude in India and Bangladesh

Subject ID

☐☐ Centre # ☐☐☐ Community# ☐☐☐ Household # ☐☐ Subject #

Subject Initials ☐☐☐ F M L

Today's date: ☐☐☐☐ year ☐☐ month ☐☐ day

1. Name:________________________ ________________________
Given name *Surname*

2. Husband's/ father's name: ________________________ ________________________
Given name *Surname*

3. National identity # or equivalent: ______________________________________ **N/A** ☐

3a) Health Card #: ☐☐☐☐☐☐☐☐☐☐ ☐☐ To be used for tracking or follow-up purposes only

→ Please fill left to right

4. DOB: ☐☐☐☐ *year* ☐☐ *month* ☐☐ *day* **OR** **Age** ☐☐ yrs

5. Sex: ☐ Female ☐ Male

6. Marital status: (check one only)

☐ Never married ☐ Currently married ☐ Common law/Living with partner
☐ Widowed ☐ Separated ☐ Divorced

7. Ethnicity: ☐☐ → (Please refer to facing page for codes)

8. Caste/Tribe: ____________________________

9. What level of formal education have you <u>completed</u>? (check highest level only)**:**

☐ None
☐ Primary
☐ Secondary/high school/higher secondary
☐ Trade School/vocational school
☐ College/University
☐ Unknown

Adult Questionnaire

Remove the Office use only boxes for all countries except Canada and Sweden

Q10 → Exclude in all countries except India and Bangladesh

Q11a → Exclude in all countries except India, Sweden and Canada

Q11c) for income document only gross income (the entire amount of income before any deductions are made) for employed subjects. In India, Poland and Bangladesh ONLY

Q11d → Exclude in all countries except South Africa

11. Occupation

Group 1: Legislators, senior officials and managers

Legislators and senior officials
Corporate managers
General managers
Businessman

Group 2: Professionals

Physical, mathematical and engineering science professionals
Life science and health professionals
Teaching professionals
Other professionals

Group 3: Technicians and associate professionals

Physical, mathematical and engineering-science associate professionals/technicians
Life science and health associate professionals/technicians
Teaching associate professionals/technicians
Other associate professionals/technicians

Group 4: Clerks

Clerks
Customer service clerks

Group 5: Service workers and shop and market sales workers

Personal and protective services workers
Models, salespersons and demonstrators

Group 6: Skilled agricultural and fishery workers

Market-oriented skilled agricultural and fishery workers
Subsistence agricultural and fishery workers

Group 7: Craft and related trade workers

Extraction and building trade workers
Metal, machinery and related trades workers
Precision, handicraft, printing and related trades workers
Other craft and related trades workers

Group 8: Plant and machine operators and assemblers

Stationary plant and related operators
Machine operators and assemblers
Drivers and mobile plant operators

Group 9: Elementary occupations

Sales and services elementary occupations
Agricultural, fishery and related labourers
Labourers in mining, construction, manufacturing and transport

Group 10: Armed forces

Armed forces

Group 11: Homemaker

Housewife/Househusband

Subject ID

☐☐ Centre # ☐☐☐ Community# ☐☐☐ Household # ☐☐ Subject #

Subject Initials ☐☐☐ F M L

10. What is the highest level of education completed by (check highest level only)**:**

- ☐ Illiterate
- ☐ Primary
- ☐ Secondary
- ☐ High school
- ☐ Higher secondary
- ☐ College/University
- ☐ Unknown

11a) During your working life, what has been or what was your main occupation?____________________________

b) Please indicate which group best describes your main occupation.

(Please refer to facing page for definitions of groups and instruction manual for detailed definitions)

☐ **Group 1** ☐ **Group 2** ☐ **Group 3** ☐ **Group 4** ☐ **Group 5**

☐ **Group 6** ☐ **Group 7** ☐ **Group 8** ☐ **Group 9** ☐ **Group 10** ☐ **Group 11**

c) How much do you earn on average in a month? ____

d) What is your main source of income? ____

If occupation is group 11 (homemaker) go to question 13

12. Are you currently employed?

☐ No → **(answer 12a - 12b)** ☐ Yes → **Go to #13**

a) Are you retired/stopped work from your primary occupation due to old age? ☐ No ☐ Yes

b) Have you stopped working due to illness? ☐ No ☐ Yes

Adult Questionnaire

Question #13- remove duration, cause and other from all countries except India and Bangladesh and Ghana.

13. Disability

Cause of Disability

1= accident
2= leprosy
3= polio
4= stroke
5= congenital
6= aging
7= Physical assault/ Violent crime
8= Armed conflict
9= Domestic violence
10= Other

KENYA:

13e) Changed to read: Do you have any trouble reading or seeing the individual grains eg. rice, sorghum, green grains on your plate (with glasses worn)?

ZIMBABWE:

13e) Exclude → Rice

13f) Exclude → Feet

IRAN and GHANA:

13e) Exclude → Corn

Exclude question 15 for all countries except India, China and Turkey

Subject ID

☐☐ Centre # ☐☐☐ Community# ☐☐☐ Household # ☐☐ Subject #

Subject Initials ☐☐☐ F M L

13. CURRENT DISABILITY:

(Please refer to facing page for disability causes)

		No	Yes	Duration (years)	Cause	Other
a)	Do you have any problems using your fingers to grasp or handle?	☐	☐ →	☐☐	☐☐	______
b)	Do you have any trouble walking about?	☐	☐ →	☐☐	☐☐	______
c)	Do you have any trouble bending down and picking up an object from the floor?	☐	☐ →	☐☐	☐☐	______
d)	Do you require a walking stick cane/walker to move about?	☐	☐ →	☐☐	☐☐	______
e)	Do you have any trouble reading or seeing the individual grains of rice/corn on your plate? (with glasses worn)	☐	☐ →	☐☐	☐☐	______
f)	Do you have trouble seeing a person from across the room? (12 feet/3.5 meters) (with glasses worn)	☐	☐ →	☐☐	☐☐	______
g)	Do you have trouble speaking and being understood?	☐	☐ →	☐☐	☐☐	______
h)	Do you have any trouble hearing what is said in a normal conversation?	☐	☐ →	☐☐	☐☐	______

Subject Medical History

14. Have you experienced any of the following in the last six months?

		No	Yes			No	Yes
a)	Chest pain or tightness with usual activity	☐	☐	i)	Vomiting	☐	☐
**If Yes,** →	does the pain spread to the back, neck or inner border of arm	☐	☐	j)	Loss of appetite	☐	☐
b)	Breathlessness with usual activity	☐	☐	k)	Painful or bleeding teeth/gums	☐	☐
c)	Cough for at least 2 weeks	☐	☐	l)	Jaundice	☐	☐
d)	Any sputum while coughing	☐	☐	m)	Burning while passing urine	☐	☐
e)	Blood in sputum	☐	☐	n)	Swelling of feet	☐	☐
f)	Wheezing or whistling in the chest	☐	☐	o)	Swelling of face	☐	☐
g)	Early morning cough with chest tightness	☐	☐	p)	Blood in urine	☐	☐
h)	Loose stools/diarrhea for at least 3 days	☐	☐	q)	Involuntary weight loss of > 3kg	☐	☐

15. Have you had cough with sputum for 3 months each year in at least the last 2 years? No ☐ Yes ☐

16 a) Do you use glasses/spectacles/contact lenses at present? No ☐ Yes ☐

b) Do you use a hearing aid? No ☐ Yes ☐

Adult Questionnaire

17. Past Medical History

**UAE**: Exclude	→	Chagas, AIDS/HIV
**Russia**: Exclude	→	Chagas, Malaria
**Chile**: Exclude	→	Chagas, Malaria
**India**: Exclude	→	Chagas
**Iran**: Exclude	→	Chagas, AIDS/HIV
**Zimbabwe**: Exclude	→	Chagas
**Bangladesh**: Exclude	→	Chagas
**Poland**: Exclude	→	Chagas
**Ghana**: Exclude	→	Chagas
**Canada:** Exclude	→	Tuberculosis, Malaria
**Exclude**	→	Chagas in all countries except South America
**Exclude**	→	Chinese medicine in all countries except Asia

Cancer Sites

1= Mouth
2= Esophagus
3= Stomach
4= Small intestine
5= Large intestine including rectum
6= Pancreas
7= Liver
8= Lung
9= Breast
10= Cervical/uterine/ovarian
11= Prostate
12= Head and neck
13= Other, specify

Subject ID

Centre #	Community#	Household #	Subject #

Subject Initials F M L

17. Have you ever been diagnosed with any of the following? (check all that apply)

	No	Yes	#of yrs since diagnosis
a) Diabetes	☐	☐	
b) Hypertension/ high blood pressure	☐	☐	
c) Stroke	☐	☐	
d) Angina/heart attack/ Coronary artery disease	☐	☐	
e) Heart failure	☐	☐	
f) Other heart disease	☐	☐	
g) Hepatitis/Jaundice	☐	☐	
h) Cancer	☐	☐	

Please refer to facing page for cancer sites — *site* ____ *other, specify* ____

	No	Yes	#of yrs since diagnosis
i) COPD	☐	☐	
j) Asthma	☐	☐	
k) Tuberculosis	☐	☐	
l) Malaria	☐	☐	
m) Chagas	☐	☐	
n) HIV/AIDS (Not answered ☐)	☐	☐	

18. Have you been taking any medications regularly (ie. at least once per week) in the last month? ☐ No → **go to 19** ☐ Yes

a) If yes, for what conditions:

	No	Yes
Blood pressure	☐	☐
Cholesterol lowering drugs	☐	☐
Stroke	☐	☐
Diabetes	☐	☐
Asthma	☐	☐
Chinese medicine	☐	☐
Others	☐	☐ → If Yes, specify ____________
Unknown	☐	☐

Page 4a) is for the Palestine only

Subject ID

☐☐	☐☐☐	☐☐☐	☐☐
Centre #	*Community#*	*Household #*	*Subject #*

Subject Initials ☐☐☐
F M L

17l) Who is your main health care provider for each of the following?

	Health care Provider		
	**MOH**	**UNRWA**	**Private**
**a)** Diabetes	☐	☐	☐
**b)** Hypertension/ high blood pressure	☐	☐	☐
**c)** Stroke	☐	☐	☐
**d)** Angina/heart attack/ Coronary artery disease	☐	☐	☐
**e)** Heart failure	☐	☐	☐
**f)** Other heart disease	☐	☐	☐
**g)** Hepatitis/Jaundice	☐	☐	☐
**h)** Cancer	☐	☐	☐
**i)** COPD	☐	☐	☐
**j)** Asthma	☐	☐	☐
**k)** Tuberculosis	☐	☐	☐

Adult Questionnaire

18b) If name of medication is unknown, please list as unknown.

Exclude Q 23a) and b) in Canada

Exclude Q 24a) and b) in Canada

Subject ID

☐☐	☐☐☐	☐☐☐	☐☐
Centre #	*Community#*	*Household #*	*Subject #*

Subject Initials ☐☐☐
F M L

18b) List all the medications you are <u>currently</u> consuming at least once a week for the last month?

i) ______________________ ii) ______________________

iii) ______________________ iv) ______________________

v) ______________________ vi) ______________________

vii) ______________________ viii) ______________________

<u>Men go to question #23</u>

<u>For Women Only (Questions 19 - 22)</u>

19. Are you currently pregnant ? ☐ No ☐ Yes → **Go to #21**

20. Do you still have periods? ☐ No → **(answer 20a)** ☐ Yes → **Go to #21**

a) How many years since you stopped menstruating? ☐☐ years

21. Have you ever used an oral/ injectable contraceptive? ☐ No ☐ Yes

22a) How many live children have you given birth to? ☐☐ Boys ☐☐ Girls

b) Did you breast feed any of your children? ☐ No ☐ Yes

23. Do you wear a helmet when riding a moped/motorcycle?

a) As a driver ☐ No ☐ Yes ☐ Not applicable

a) As a passenger ☐ No ☐ Yes ☐ Not applicable

24. Do you wear a seatbelt when riding in a car/jeep?

a) As a driver ☐ No ☐ Yes ☐ Not applicable

a) As a passenger ☐ No ☐ Yes ☐ Not applicable

Page 5a) is for the Palestine only

Medication purchased from codes:

1 = MoH Clinic

2= UNRWA clinic pharmacy

3= Private pharmacy

4= NGO clinic pharmacy

5= Other

Subject ID

Centre #	*Community#*	*Household #*	*Subject #*

Subject Initials *F M L*

18c) For all medications listed in question 18b), please provide the following:

Medication name	Where was it purchased (see codes on facing page) if code 5 please specify	How much was paid (NIS)	Date purchased year	Date purchased month	How many days does medication last
i)					
ii)					
iii)					
iv)					
v)					
vi)					
vii)					
viii)					

	No	Yes	N/A
**18d)** Do you sometimes forget to take your medication?	☐	☐	☐
**18e)** Do you ever feel hassled about sticking to your treatment plan?	☐	☐	☐
**18f)** When you feel like your symptoms are under control, do you sometimes stop taking your medicine?	☐	☐	☐
**18g** Have you ever cut back or stopped taking your medicine without telling your doctor, because you felt worse when you took it?	☐	☐	☐
**18h)** In the past week, did you skip any of your medication?	☐	☐	☐

Adult Questionnaire

25. Accidents and Injuries

Location of Injury

1= Factory/industrial place
2= Office
3= Agriculture field/farm
4= Home
5= Road
6= Sport/game e.g. track, court, field, etc.
7= Public building
8= Mine/quarry
9= Construction site e.g. building, road-works, etc.
10 = Other

Type of Injury

1= Burns
2= Scalds
3= Fractures
4= Muscle and ligament sprains/tears
5= Cuts and lacerations
6= Bruises and abrasions
7= Suffocation
8= Head injury (where person did not lose consciousness)
9= Head injury (where person lost consciousness for some time)

Subject ID

Centre #	Community#	Household #	Subject #

Subject Initials F M L

25. **During the past 12 months, have you had any injuries that were serious enough to limit your normal activities?** (check all that apply)

☐ No → **Go to #26** ☐ Yes → **(answer 25a - 25s)**

If yes, please provide details:

Please refer to facing page for Location and Type Codes

**Cause of injury**			Location	Type	Absence from work or usual activities (Days)
**a)** Motor vehicle accident (as a passenger)	☐ No	☐ Yes →			
**b)** Motor vehicle accident (as a pedestrian)	☐ No	☐ Yes →			
**c)** Struck by an object	☐ No	☐ Yes →			
**d)** Explosion	☐ No	☐ Yes →			
**e)** Natural/environmental factors (gales/cyclones/lightning, etc.)	☐ No	☐ Yes →			
**f)** Suffocation	☐ No	☐ Yes →			
**g)** Poisoning	☐ No	☐ Yes →			
**h)** Snake/scorpion bite	☐ No	☐ Yes →			
**i)** Fall	☐ No	☐ Yes →			
**j)** Fire/flames, resultant fumes	☐ No	☐ Yes →			
**k)** Physical assault (gun, kidnapping, etc.)/violent crime	☐ No	☐ Yes →			
**l)** Domestic violence (beaten by a family member)	☐ No	☐ Yes →			
**m)** Drowning/submersion	☐ No	☐ Yes →			
**n)** Hot or corrosive liquids/floods/substances	☐ No	☐ Yes →			
**o)** Crush injuries (boulders, building materials, etc.)	☐ No	☐ Yes →			
**p)** Accident caused by machinery	☐ No	☐ Yes →			
**q)** Attempted suicide	☐ No	☐ Yes →			
**r)** Armed conflict	☐ No	☐ Yes →			
**s)** Other(specify) ______________	☐ No	☐ Yes →			

Adult Questionnaire

Location of Fractures

1= Hip/pelvis
2= Thigh
3= Leg
4= Forearm
5= Wrist
6= Hand/finger
7= Vertebrae (back)
8= Other

Fractures: In situations where subjects are in a cast and cannot differentiate between ligament tear or fracture, include as fracture only if doctor confirmed it as a broken bone

27c) Tobacco: Regular use is defined as consuming at least one tobacco product per day.

27c. History of tobacco use:

Rolled tobacco leaves is specific to China only, remove from all other countries.

Exclude Opium in all countries except Iran.

**China:**	Exclude →	Chewing Tobacco, Snuff and Beedies
**UAE:**	Exclude →	Chewing Tobacco, Snuff and Beedies
**Russia:**	Exclude →	Chewing Tobacco, Snuff and Beedies, Sheesha/Waterpipe Hooka
**Chile:**	Exclude →	Chewing Tobacco, Snuff and Beedies, Sheesha/Waterpipe Hooka
**Argentina:**	Exclude →	Snuff and Beedies, Sheesha/Waterpipe Hooka
**Iran:**	Exclude →	Snuff
**Zimbabwe:**	Exclude →	Beedies, Cigars and Sheesha/Waterpipe Hooka
**Bangladesh:**	Exclude →	Cigars, Pipes, snuff and opium
**Poland:**	Exclude →	Beedies, Sheesha/Watherpipe Hooka, Rolled Tobbaco leaves, Dagga
**Turkey:**	Exclude →	Beedies, Snuff, opium, chewing tobacco, Rolled Tobbaco leaves, Dagga
**Ghana:**	Exclude →	Beedies, Sheesha/waterpipe Hookah, Dagga

Duration of use:

For those that have consumed tobacco for <1 year, please enter “0”

Subject ID

Centre #	Community#	Household #	Subject #
☐☐	☐☐☐	☐☐☐	☐☐

Subject Initials ☐☐☐
F M L

26. Have you ever fractured a bone? ☐ No (go to #27) ☐ Yes (if yes, answer a),b) and c)

a) Number of fractures ☐☐

b) Years since last fracture ☐☐ (yrs)

c) Bone (s) broken in the most recent fracture(if more than 3, list most severe sites)

Please refer to facing page for fracture locations

(location) If other, specify
☐ → ______________________
☐ → ______________________
☐ → ______________________

Tobacco

27. Which best describes your history of tobacco use?

a) ☐ Formerly used tobacco products ☐ Currently use tobacco products ☐ **Never used tobacco products** → **Go to #28**

b) At what age did you start? ☐☐ yrs

c) Have you ever regularly used any of the following tobacco products? (check all that apply)

	Average amount/day		Duration (years)	Past users only: When Stopped (years ago)	Past users only: If less than 1 yr (months ago)
**(i)** Cigarettes (all kinds)	☐☐☐	number	☐☐	☐☐	☐☐
**(ii)** Beedies	☐☐☐	number	☐☐	☐☐	☐☐
**(iii)** Cigars	☐☐☐	number	☐☐	☐☐	☐☐
**(iv)** Pipes	☐☐☐	number	☐☐	☐☐	☐☐
**(v)** Sheesha/water pipe Hookah	☐☐☐	# of times	☐☐	☐☐	☐☐
**(vi)** Chewing tobacco	☐☐☐	# of times	☐☐	☐☐	☐☐
**(vii)** Snuff	☐☐☐	# of times	☐☐	☐☐	☐☐
**(viii)** Opium	☐☐☐	# of times	☐☐	☐☐	☐☐
**(ix)** Rolled tobacco leaves	☐☐☐	number	☐☐	☐☐	☐☐
**(x)** Dagga	☐☐☐		☐☐	☐☐	☐☐
**(xi)** Other ________ *Specify*	☐☐☐		☐☐	☐☐	☐☐

29. Betel nut/paan

Exclude in → Chile, China, UAE, Russia, Argentina, Colombia, Zimbabwe and South Africa, Poland, Turkey and Ghana

29c) Betel nut/paan: Regular use is defined as at least once per week

Subject ID

☐☐ Centre # ☐☐☐ Community# ☐☐☐ Household # ☐☐ Subject #

Subject Initials ☐☐☐ F M L

Question 28 to be answered by non-smokers and former smokers only

28. During the past 12 months, have you been regularly (at least once per week) exposed to other people's tobacco smoke?
("Exposed" is defined as a minimum of 5 consecutive minutes, during which you inhale other people's smoke.)

☐ No → **Go to #29** ☐ Yes → Please answer questions **28a**

a) Over the past 12 months, what has been your typical exposure to other peoples smoke?
("Exposed" is defined as a minimum of 5 consecutive minutes, during which you inhale other peoples smoke)

Select ONE only

☐ 1-2 times/week ☐ 3-6 times/week ☐ at least once a day ☐ 2-3 times/day ☐ 4 or more times/day

29. Which best describes your history of Betel Nut use?

a) ☐ Formerly used betel nut products ☐ Currently use betel nut products ☐ **Never used betel nut products** → **Go to #30**

b) At what age did you start? ☐☐ yrs

c) Have you ever regularly used betel nut in the following forms?(check all that apply)

			Past users only	
	**Average amount/day**	**Duration** (years)	**When Stopped** (years ago)	**If less than 1 yr** (months ago)
Paan with betel Nut	☐☐☐ # of times	☐☐	☐☐	☐☐
Betel Nut	☐☐☐ # of times	☐☐	☐☐	☐☐
Tobacco added to Paan	☐☐☐ # of times	☐☐	☐☐	☐☐

Adult Questionnaire

30c) Alcoholic Beverage: Regular use is defined as at least once a month**.**

Spirits includes RAKI 40-50% for Turkey

30a. Alcoholic Beverage

Bangladesh:

Exclude ⟶ wine, beer, liquor >38% and liquor <38%

CHINA:

Exclude ⟶ Spirits(rum, whisky, gin, vodka etc.), Country Liquor/arrak/sugar cane spirit, wine 60ml

RUSSIA and ARGENTINA:

Exclude ⟶ Liquor <38%, Liquor >38%, wine 125ml

All Other Countries:

Exclude ⟶ Liquor <38%, Liquor >38% and Wine 60ml

Subject ID

☐☐ Centre # ☐☐☐ Community# ☐☐☐ Household # ☐☐ Subject #

Subject Initials ☐☐☐ F M L

30. Which best describes your history of alcohol use?

a) ☐ Formerly used alcohol products ☐ Currently use alcohol products ☐ **Never used alcohol products** → **Go to #31**

b) At what age did you start? ☐☐ yrs

c) What forms of alcohol have you regularly used? (check all that apply)

**Form of Alcohol**	**Approx. size of one "drink"**	**Frequency** Daily	Weekly	Monthly	**Average # of drinks**	**Duration** (years)	**Past users only When Stopped** (years ago)
**(i)** Spirits(rum,whisky, gin,vodka etc)	30ml	☐	☐	☐	☐☐	☐☐	☐☐
**(ii)** Wine	125ml	☐	☐	☐	☐☐	☐☐	☐☐
**(iii)** Liquor <38%	125ml	☐	☐	☐	☐☐	☐☐	☐☐
**(iv)** Liquor >38%	125ml	☐	☐	☐	☐☐	☐☐	☐☐
**(v)** Wine	60ml	☐	☐	☐	☐☐	☐☐	☐☐
**(vi)** Beer	375ml	☐	☐	☐	☐☐	☐☐	☐☐
**(vii)** Country liquor/arrack/ sugar cane spirit	30ml	☐	☐	☐	☐☐	☐☐	☐☐

d) **At least once a month, do you consume >5 alcoholic drinks/day?** ☐ No → **Go to #31** ☐ Yes

If yes,(i,ii)

i) How many times per month do you consume >5 alcoholic drinks in a day? ☐☐

ii) What is the average number of drinks that you consume each time? ☐☐

31 a) During your longest or nocturnal sleep period, what time do you normally go to bed? ☐☐ : ☐☐ (00:00-23:59)

b) During your longest or nocturnal sleep period, what time do you normally wake up? ☐☐ : ☐☐ (00:00-23:59)

c) Do you usually take naps/siestas? ☐ No ☐ Yes *Total nap duration* → ☐☐☐ mins

Adult Questionnaire

32. Are you a member of any of the following:

China: Exclude → Question #32

Russia: Exclude → Self help group

34a)

Iran: Exclude → Flour

34b)

Iran and Bangladesh: Exclude → Alcohol

35. <u>Civic organization</u>: are defined as non-profit, voluntary organization societies, self help groups and clubs.

<u>Religious organization</u>: are defined as different types of formal and informal groups set up on a religious basis.

Remove family members and other from all countries except Zimbabwe and Ghana

Subject ID

Centre # | Community# | Household # | Subject #

Subject Initials F M L

32. Are you a member of any of the following:

How often do you participate in the activities of this group?

			Per Month	OR	Per Year
**(i)** Self help group, Co-operative, Social club, Sports club,	☐ No	☐ Yes →	☐☐		☐☐
**(ii)** Religious Group (e.g: church group, etc.)	☐ No	☐ Yes →	☐☐		☐☐
**(iii)** Other ______________ *Specify*	☐ No	☐ Yes →	☐☐		☐☐

33. Please answer the following: (choose only one option for each)

	Strongly Disagree	Somewhat Disagree	Somewhat Agree	Strongly Agree
(i) People are generally honest and want to help others.	☐	☐	☐	☐
(ii) If I do nice things for someone, I can anticipate that they will respect me and treat me just as well as I treat them.	☐	☐	☐	☐

34a) The television, radio, newspaper or magazine advertisements help me decide to buy the type of: (choose only one option for each)

					Not Applicable
(i) Cooking oil	☐	☐	☐	☐	☐
(ii) Flour	☐	☐	☐	☐	☐
(iii) Rice/ Maize meal/bulgur/macaroni/spaghetti	☐	☐	☐	☐	☐

b) The television, radio, newspaper or magazine advertisements influence whether I buy: (choose only one option for each)

(i) Soft drinks	☐	☐	☐	☐	☐
(ii) Snacks	☐	☐	☐	☐	☐
(iii) Cigarettes	☐	☐	☐	☐	☐
(iv) Alcohol	☐	☐	☐	☐	☐

35. In a difficult situation, whose help can you count on from?(Please see facing page for definitions)

(i) Civic organizations: specify ______________________________

☐ none ☐ little ☐ moderate/average ☐ a great deal

(ii) Religious organizations:specify ______________________________

☐ none ☐ little ☐ moderate/average ☐ a great deal

(iii) Family members:

☐ none ☐ little ☐ moderate/average ☐ a great deal

(iv) Other: specify ______________________________

☐ none ☐ little ☐ moderate/average ☐ a great deal

Subject ID

☐☐	☐☐☐	☐☐☐	☐☐
Centre #	*Community#*	*Household #*	*Subject #*

Subject Initials ☐☐☐
F M L

36. Have you experienced any of the following events during the last 12 months?

	No response	No	Yes	
**(i)** Loss of job	☐	☐	☐	
**(ii)** Retirement	☐	☐	☐	
**(iii)** Loss of crop/business failure	☐	☐	☐	
**(iv)** Household break in	☐	☐	☐	
**(v)** Marital separation/divorce	☐	☐	☐	
**(vi)** Other major intra-family conflict	☐	☐	☐	→ Please specify ____________________
**(vii)** Major personal injury or illness	☐	☐	☐	
**(viii)** Violence	☐	☐	☐	
**(ix)** Armed conflict/war	☐	☐	☐	
**(x)** Death of a spouse	☐	☐	☐	
**(xi)** Death/major illness of another close family member	☐	☐	☐	
**(xii)** Other major stress	☐	☐	☐	→ Please specify ____________________
**(xiii)** Wedding of family member	☐	☐	☐	
**(xiv)** New job	☐	☐	☐	
**(xv)** Birth in the family	☐	☐	☐	
**(xvi)** Seperation from family	☐	☐	☐	
**(xvii)** Unavailability of food/ food insecurity	☐	☐	☐	

Subject ID

☐☐ Centre # ☐☐☐ Community# ☐☐☐ Household # ☐☐ Subject #

Subject Initials ☐☐☐ F M L

37. Please answer the following: (Choose only one option for each)

For the following question, stress is defined as feeling irritable or filled with anxiety, or as having sleeping difficulties as a result of conditions at work or at home.

	No response	Never Experienced Stress	Some Period of Stress	Several Periods of Stress	Permanent Stress
**a)** How often have you felt stress at work in the last 12 months? (Mark here if not applicable: i.e. no longer working ☐)	☐	☐	☐	☐	☐
**b)** How often have you felt stress at home in the last 12 months?	☐	☐	☐	☐	☐

38. What level of financial stress have you felt in the last 12 months?

☐ No response ☐ Little/none ☐ Moderate ☐ High/severe

39. During the past twelve months, was there ever a time when you felt sad, blue, or depressed for two weeks or more in a row?

☐ No ☐ Yes → **If yes, during those times, did you:**

	No response	No	Yes
**a)** Lose interest in most things like hobbies, work or activities that usually give you pleasure?	☐	☐	☐
**b**) Feel tired or low on energy?	☐	☐	☐
**c)** Gain or lose weight?	☐	☐	☐
**d)** Have more trouble falling asleep than you usually do?	☐	☐	☐
**e)** Have more trouble concentrating than usual?	☐	☐	☐
**f)** Think a lot about death (either your own, someone else's, or death in general)	☐	☐	☐
**g)** Feel down on yourself, no good or worthless?	☐	☐	☐

Exclude Q40 in Canada

Subject ID

Centre # | Community# | Household # | Subject #

Subject Initials F M L

40. Please answer the following: (Choose only one option for each)

	Strongly Disagree	Somewhat Disagree	Somewhat Agree	Strongly Agree
**a)** I can do most of my regular shopping (food, household necessities, etc.) at stores within easy walking distance (less than 15 minutes) of my home.	☐	☐	☐	☐
**b)** Walking or bicycling in my neighbourhood is difficult because of the speed and/or amount of traffic.	☐	☐	☐	☐
**c)** My neighbourhood is generally free from pollution (litter, air pollution and noise pollution).	☐	☐	☐	☐
**d)** My neighbourhood streets are well lit at night.	☐	☐	☐	☐
**e)** I can see other people when I am walking in my neighbourhood.	☐	☐	☐	☐
**f)** I can speak to other people when I am walking in my neighbourhood.	☐	☐	☐	☐
**g)** There is a high crime rate in my neighbourhood.	☐	☐	☐	☐
**h)** There is a problem with unattended dogs in my neighbourhood.	☐	☐	☐	☐

Exclude contents of page 13a) for all countries except Canada

Subject ID

☐☐	☐☐☐	☐☐☐	☐☐
Centre #	*Community#*	*Household #*	*Subject #*

Subject Initials ☐☐☐
F M L

40d) Please answer the following: (choose only one option for each)

	**Definitely false**	**Mostly false**	**Mostly true**	**Definitely true**
(i) When I smell a sizzling steak or juicy piece of meat, I find it very difficult to keep from eating, even if I have just finished a meal.	☐	☐	☐	☐
(ii) I deliberately take small helpings as a means of controlling my weight.	☐	☐	☐	☐
(iii) When I feel anxious, I find myself eating.	☐	☐	☐	☐
(iv) Sometimes when I start eating, I just can't seem to stop.	☐	☐	☐	☐
(v) Being with someone who is eating often makes me hungry enough to eat also.	☐	☐	☐	☐
(vi) When I feel blue, I often overeat.	☐	☐	☐	☐
(vii) When I see a real delicacy, I often get so hungry that I have to eat right away.	☐	☐	☐	☐
(viii) I get so hungry that my stomach often seems like a bottomless pit.	☐	☐	☐	☐
(ix) I am always hungry so it is hard for me to stop eating before I finish the food on my plate.	☐	☐	☐	☐
(x) When I feel lonely, I console myself by eating.	☐	☐	☐	☐
(xi) I consciously hold back at meals in order not to gain weight.	☐	☐	☐	☐
(xii) I do not eat some foods because they make me fat.	☐	☐	☐	☐
(xiii) I am always hungry enough to eat at any time.	☐	☐	☐	☐

40e) How often do you feel hungry? (check one only)**:**

☐ only at meal times ☐ sometimes between meals ☐ often between meals ☐ almost always

40f) How frequently do you avoid "stocking up" on tempting foods? (check one only)**:**

☐ almost never ☐ seldom ☐ usually ☐ almost always

Exclude contents of page 13b) for all countries except Canada

Subject ID

☐☐ Centre # ☐☐☐ Community# ☐☐☐ Household # ☐☐ Subject #

Subject Initials ☐☐☐ F M L

40g) How likely are you to consciously eat less than you want? (check one only)**:**

☐ unlikely ☐ slightly unlikely ☐ moderately likely ☐ very likely

40h) Do you go on eating binges though you are not hungry? (check one only)**:**

☐ never ☐ rarely ☐ sometimes ☐ at least once a week

40i) On a scale of 1 to 8, where 1 means no restraint in eating (eating whatever you want, whenever you want it) and 8 means total restraint (constantly limiting food intake and never "giving in"), what number would you give yourself?

☐ **1** ☐ **2** ☐ **3** ☐ **4** ☐ **5** ☐ **6** ☐ **7** ☐ **8**

**40j) Does cost prevent you from:** (choose only one option for each)	**Strongly Disagree**	**Somewhat Disagree**	**Somewhat Agree**	**Strongly Agree**
a**)** Getting enough exercise	☐	☐	☐	☐
b) Eating enough:				
(i) fruits and vegetables	☐	☐	☐	☐
(ii) fish	☐	☐	☐	☐
(iii) meat	☐	☐	☐	☐

40k) Do you consume the following foods because they are inexpensive?
(choose only one option for each)

(i) chips	☐	☐	☐	☐
(ii) soda	☐	☐	☐	☐
(iii) cookies	☐	☐	☐	☐

Exclude contents of page 14 for all countries except South Africa, Bangladesh and Turkey

Subject ID

☐☐ Centre # ☐☐☐ Community# ☐☐☐ Household # ☐☐ Subject #

Subject Initials ☐☐☐ F M L

40a) Please answer the following: (Please check all that apply)

i) Has your household been a victim of the following crime(s) in the last 12 months?

	No	Yes
1. Armed robbery	☐	☐
2. Violent attacks	☐	☐
3. Murder	☐	☐
4. Vehicle hijacking	☐	☐
5. House breaking	☐	☐
6. Theft	☐	☐
7. Rape	☐	☐
8. Women abuse eg. (beat,swear-words,sexual) please specify ______________________	☐	☐
9. Child abuse eg. (burn,swear-words,rejection) please specify ______________________	☐	☐
10. Child sexual abuse	☐	☐
11. Other, please specify ______________________	☐	☐

ii) Do you think that crime in your area has increased in the past 5 years? ☐ No ☐ Yes

if yes, which of the following crime(s)?

- ☐ Armed robbery
- ☐ Violent attacks
- ☐ Murder
- ☐ Vehicle hijacking
- ☐ House breaking
- ☐ Theft
- ☐ Rape
- ☐ Women abuse
- ☐ Child abuse
- ☐ Child sexual abuse
- ☐ Other, please specify ______________

Exclude contents of page 15 for all countries except South Africa and Bangladesh

Subject ID

☐☐ *Centre #* ☐☐☐ *Community#* ☐☐☐ *Household #* ☐☐ *Subject #*

Subject Initials ☐☐☐ *F M L*

40b) Questions on HIV:

i) Do you know people who have HIV/AIDS? ☐ No ☐ Yes

if yes, which of these people: (please mark all that apply)

- ☐ Your children
- ☐ Your grandchildren
- ☐ Your spouse
- ☐ Your family members
- ☐ Your friends
- ☐ People in the community

ii) What would you consider the mean age of the people who are ill/have died of HIV/AIDS?

☐ Younger than 10 years ☐ Between 11-20 years ☐ Between 21-30 years

☐ Between 31-40 years ☐ Between 41-50 years ☐ Over 50 years

iii) If someone in your household is HIV positive, who is the primary caregiver?

- ☐ Spouse
- ☐ Parents
- ☐ Family member
- ☐ Child.children
- ☐ Friends
- ☐ Volunteer

40c) Do you care for any orphans in your family? ☐ No ☐ Yes

Adult Questionnaire

41. Remove question 41 in Canada

42b) Health History:

Cancer Sites

1= Mouth
2= Esophagus
3= Stomach
4= Small intestine
5= Large intestine including rectum
6= Pancreas
7= Liver
8= Lung
9= Breast
10= Cervical/uterine/ovarian
11= Prostate
12= Head and neck
13= Other, specify

Subject ID

☐☐	☐☐☐	☐☐☐	☐☐
Centre #	*Community#*	*Household #*	*Subject #*

Subject Initials ☐☐☐
F M L

41. How long would it take you to get from your house to the nearest facility <u>if you walked</u>?

	Minutes	Don't know		Minutes	Don't know
i) grocery/convenience store	☐☐☐	☐	iv) video store	☐☐☐	☐
ii) bank	☐☐☐	☐	v) non-fast food restaurant	☐☐☐	☐
iii) post office	☐☐☐	☐	vi) fast food restaurant	☐☐☐	☐

42a) Total number of siblings ☐☐

b) Health History: Complete for all parents and siblings, alive or dead

	Father			Mother			Siblings			# of siblings with the condition
	**Unknown**	**No**	**Yes**	**Unknown**	**No**	**Yes**	**Unknown**	**No**	**Yes**	
Diabetes	☐	☐	☐	☐	☐	☐	☐	☐	☐	**If yes** → ☐☐
Coronary Heart Disease	☐	☐	☐	☐	☐	☐	☐	☐	☐	☐☐
High Blood Pressure	☐	☐	☐	☐	☐	☐	☐	☐	☐	☐☐
Stroke	☐	☐	☐	☐	☐	☐	☐	☐	☐	☐☐
Cancer **Please refer to facing page for cancer sites**	☐	☐	☐	☐	☐	☐	☐	☐	☐	☐☐
→ **if Yes, indicate site**	☐☐			☐☐			☐☐			
	____ Other, Specify			____ Other, Specify			____ Other, Specify			

Adult Questionnaire

If subject refuses to provide any of the measures, enter a value of "0" into each of the boxes for that question

For more detailed instructions please refer to the instruction manual

Exclude all skinfold questions from all countries except India and Bangladesh

Exclude upper flexed arm circumference from all countries except South Africa

Subject ID

☐☐ Centre # ☐☐☐ Community# ☐☐☐ Household # ☐☐ Subject #

Subject Initials ☐☐☐ F M L

43. Physical Measurements

a) Sitting Right arm blood pressure

#1 ☐☐☐ *Systolic* ☐☐☐ *Diastolic* **mmHg**

#2 ☐☐☐ *Systolic* ☐☐☐ *Diastolic* **mmHg**

b) Heart Rate

#1 ☐☐☐ **beats/min**

#2 ☐☐☐ **beats/min**

c) Waist

#1 ☐☐☐.☐ **cm**

#2 ☐☐☐.☐ **cm**

→ ☐ minimal/no clothing ☐ full clothing

d) Weight ☐☐☐.☐ **kg** → ☐ minimal/no clothing ☐ full clothing

e) Hip

#1 ☐☐☐.☐ **cm**

#2 ☐☐☐.☐ **cm**

→ ☐ minimal/no clothing ☐ full clothing

f) Height ☐☐☐.☐ **cm** (without shoes)

44a) Circumference of mid upper right arm: ☐☐.☐ **cm**

b) Circumference of right calf: ☐☐.☐ **cm**

c) Head Circumference: ☐☐☐.☐ **cm**

d) Upper flexed arm circumference ☐☐.☐ **cm**

e) Circumference of right thigh ☐☐.☐ **cm**

45a) Right arm triceps skinfold:

#1 ☐☐☐.☐ **mm**

#2 ☐☐☐.☐ **mm**

#3 ☐☐☐.☐ **mm**

b) Right calf skinfold:

#1 ☐☐☐.☐ **mm**

#2 ☐☐☐.☐ **mm**

#3 ☐☐☐.☐ **mm**

Exclude supra spinal skinfolds, humerous and femur breadth from all countries except South Africa

Subject ID

Centre #	Community#	Household #	Subject #
☐☐	☐☐☐	☐☐☐	☐☐

Subject Initials ☐☐☐ *F M L*

c) Biceps skinfold

#1 ☐☐☐.☐ **mm**
#2 ☐☐☐.☐ **mm**
#3 ☐☐☐ ☐ **mm**

d) Subscapular skinfold

#1 ☐☐☐.☐ **mm**
#2 ☐☐☐.☐ **mm**
#3 ☐☐☐ ☐ **mm**

e) Supra spinal skinfolds

#1 ☐☐☐.☐ **mm**
#2 ☐☐☐.☐ **mm**
#3 ☐☐☐ ☐ **mm**

46 a) Humerous breadth ☐☐.☐ **cm**

b) Femur breadth ☐☐.☐ **cm**

47. Grip Strength (Maximal contraction):

a) Non-dominant hand:

#1 ☐☐ **kg.**
#2 ☐☐ **kg.**
#3 ☐☐ **kg.**

b) Dominant hand:

#1 ☐☐ **kg.**
#2 ☐☐ **kg.**
#3 ☐☐ **kg.**

Adult Questionnaire

If subject refuses to provide any of the measures, enter a value of “0” into each of the boxes for that question

For more detailed instructions please refer to the instruction manual

48. Spirometry:

American Thoracic Society criteria for acceptable spirograms:
Spirograms are acceptable if they are free from:

1. Cough during exhalation
2. Early termination or cut-off
3. Variable effort
4. Leaks
5. Obstructed mouth piece

Subject ID

☐☐ Centre # ☐☐☐ Community# ☐☐☐ Household # ☐☐ Subject #

Subject Initials ☐☐☐ F M L

48. Spirometry:

a) FEV1 (Litre): **#1** ☐.☐☐ **#2** ☐.☐☐ **#3** ☐.☐☐

b) Does FEV1 obtained meet ATS criteria?

☐ No ⟶ (answer (i) to (iii) ☐ Yes ⟶ Go to **c)**

Reasons for not meeting the ATS criteria: (check all that apply)

i) Cough ☐

ii) Values not within 0.2L of each other ☐

iii) Less than 3 values ☐

c) FVC (Litre): **#1** ☐.☐☐ **#2** ☐.☐☐ **#3** ☐.☐☐

d) Does FVC obtained meet ATS criteria?

☐ No ⟶ (answer (i) to (iii) ☐ Yes ⟶ Go to **e)**

Reasons for not meeting the ATS criteria: (check all that apply)

i) Cough ☐

ii) Values not within 0.2L of each other ☐

iii) Less than 3 values ☐

e) PEFR (Litre/min): **#1** ☐☐☐ **#2** ☐☐☐ **#3** ☐☐☐

f) Does PEFR obtained meet ATS criteria?

☐ No ⟶ (answer (i) to (ii) ☐ Yes ⟶ Go to **Q#49**

Reasons for not meeting the ATS criteria: (check all that apply)

i) Cough ☐

ii) Less than 3 values ☐

Exclude question 49 in all countries except India and Bangladesh

Subject ID

☐☐	☐☐☐	☐☐☐	☐☐
Centre #	*Community#*	*Household #*	*Subject #*

Subject Initials ☐☐☐
F M L

49. Describe each eye:

	Normal	Blindness due to causes other than cataracts	Immature cataract	Mature cataract	Operated due to cataract				
**Right eye**	☐	☐	☐	☐	☐	*how long ago?* →	☐☐ **YY**	**or**	☐☐ **MM**
**Left eye**	☐	☐	☐	☐	☐	*how long ago?* →	☐☐ **YY**	**or**	☐☐ **MM**

50. ECG obtained? No ☐ → **Go to #51** Yes ☐

a) **2 0** ☐☐ ☐☐ ☐☐ → Place ECG :File Label Here
year *month* *day*

b) **Please print ECG label #:** ☐☐☐☐☐☐

51 a) Blood sample obtained? No ☐ → **Go to #52** Yes ☐

b) ☐ Fasting sample ☐ Non-fasting sample

c) **2 0** ☐☐ ☐☐ ☐☐ **Time** ☐☐ **:** ☐☐ → **Hours since any food/beverage consumed (excluding water)** ☐☐
year *month* *day* (00:00-23:59)

d) **Please print Blood label #:** ☐☐☐☐☐☐

Place Blood label here

52 a) Urine sample obtained? No ☐ → **Go to #53** Yes ☐

b) ☐ Fasting sample ☐ Non-fasting sample

c) **Please print Urine label #:** ☐☐☐☐☐☐

Place Urine label here

53. Name of Interviewer: ______ ____________________ **Interviewer Code:** ☐☐☐
(please print) *First Initial* *Last Name*

Page 21 for use in Canada, Palestine, Tanzania, Saudi Arabia, Philippines

Subject ID

Centre #	Community#	Household #	Subject #

Subject Initials F M L

BIA MEASUREMENTS

a) **Total Body Weight** ☐☐☐.☐ **kgs**

b) **Body fat %** ☐☐.☐

c) **Body water %** ☐☐.☐

d) **Muscle mass** ☐☐☐.☐

e) **Physical rating** ☐

f) **BMR** ☐☐☐☐

g) **Metabolic age** ☐☐

h) **Bone** ☐☐.☐

i) **Visceral fat** ☐☐

Page 22a for Saudi Arabia only

Subject ID

Centre #	*Community#*	*Household #*	*Subject #*

Subject Initials *F M L*

55. Please answer the following: (Choose only one option for each)

What is main reason for not exercising:

a) Bad weather ☐

b) Lack of transportation to walking areas
(unable to drive in female etc) ☐

c) Health barriers ☐

d) Absence of walking/ sport facilities ☐

e) Busy lifestyle (not enough time) ☐

f) Not interested ☐

g) Other ______________________ ☐
Please specify

Page 22b for Palestine only

Subject ID

☐☐ Centre # ☐☐☐ Community# ☐☐☐ Household # ☐☐ Subject #

Subject Initials ☐☐☐ F M L

54. Do you take medications? If yes, please complete the questions below. ☐ **No** ☐ **Yes**

	**No**	**Yes**
a) Do you sometimes forget to take your medicine?	☐	☐
b) In the past 2 weeks, were there any days when you did not take your medicine for a reason other than forgetting?	☐	☐
c) Have you ever cut back or stopped taking your medicine, without telling your doctor, because you felt worse when you took it?	☐	☐
d) When you travel or leave home, do you sometimes forget to bring along your medicine?	☐	☐
e) Did you take all of your medicine yesterday?	☐	☐
f) When you feel like your symptoms are under control, do you sometimes stop taking your medicine?	☐	☐
g) Do you ever feel hassled about sticking to your treatment plan?	☐	☐

h) How often do you have difficulty remembering to take all your medicine?

☐ Never/rarely ☐ Once in awhile ☐ Sometimes ☐ Usually ☐ All the time

55. Do you have health insurance? ☐ **No** ☐ **Yes**

If yes, please complete a) and b)

a) What is the insurance that you use the most?

☐ MoH ☐ Military ☐ UNRWA ☐ Social Affair/Elderly ☐ Intifada Al-Aqsa

☐ Private ☐ Israeli ☐ Outside the country insurance

b) Is your family covered by the insurance? ☐ No ☐ Yes

We are very grateful to you for your participation in this study. All information given by you will be held in strict confidence, and will be used for the purpose of this study only after removing any personal identifying information.

Physical Activity Questionnaire

INSTRUCTIONS

Please answer EACH question by marking an X in ONE BOX on each line: (unless otherwise instructed)

[X]

OR

By writing number(s) in the spaces provided:

[1] [8]

OR

By specifying the answer on the line(s) provided

July, 2007

Physical Activity Questionnaire

Subject Initials- F= first letter of first name
M= first letter of middle name
L= first letter of last name

Subject ID

☐☐ Centre # ☐☐☐ Community # ☐☐☐ Household # ☐☐ Subject #

Subject Initials ☐☐☐ *F M L*

Today's date: ☐☐☐☐ *year* ☐☐ *month* ☐☐ *day*

1. During your working life, what has been or what was your main occupation?

____

Part 1: Job-related physical activity

(paid or unpaid jobs outside of the home) *Do not include house or yard work*

2. Do you currently have a job or do any unpaid work outside your home?

☐ No ⟶ **If no, Go to PART 2** ☐ Yes ⟶ **If yes, Go to question # 3**

3. During the *last 7 days*, on how many days did you do *heavy* physical actitivies for at least 10 min (eg. heavy lifting, digging, heavy construction) as part of your work?

☐ Days per week ⟶ Usual time spent on one of those days doing heavy physical activities at work ☐☐ hrs. ☐☐ min.

☐ No vigorous activity at work

4. During the *last 7 days*, on how many days did you do *moderate* physical activities for at least 10 min (eg. carrying light loads) as part of your work? *Please do not include walking*

☐ Days per week ⟶ Usual time spent on one of those days doing moderate physical activities at work ☐☐ hrs. ☐☐ min.

☐ No moderate activity at work

5. During the *last 7 days*, on how many days did you *walk* for at least 10 min at a time as part of your work? *Please do not count any walking you did to travel to or from work*

☐ Days per week ⟶ Usual time spent on one of those days walking at work ☐☐ hrs. ☐☐ min.

☐ No walking at work

Subject ID

☐☐ ☐☐☐ ☐☐☐ ☐☐
Centre # *Community #* *Household #* *Subject #*

Subject Initials ☐☐☐
F M L

Part 2: Transportation related physical activity

6. During the *last 7 days*, on how many days did you *travel in a motor vehicle* to places like work, stores, movies, and so on?

☐ Days per week → Usual time spent on one of those days traveling in a motor vehicle ☐☐ hrs. ☐☐ min.

☐ No traveling in a motor vehicle

7. During the *last 7 days*, on how many days did you *bicycle* for at least 10 minutes at a time to go from place to place?

☐ Days per week → Usual time spent on one of those days bicycling from place to place ☐☐ hrs. ☐☐ min.

☐ No bicycling from place to place

8. During the *last 7 days*, on how many days did you *walk* for at least 10 min at a time to go from place to place?

☐ Days per week → Usual time spent on one of those days walking from place to place ☐☐ hrs. ☐☐ min.

☐ No walking from place to place

Subject ID

☐☐ Centre # ☐☐☐ Community # ☐☐☐ Household # ☐☐ Subject #

Subject Initials ☐☐☐ F M L

Part 3: Housework, house maintenance, and caring for family

9. During the *last 7 days*, on how many days did you do *vigorous* physical activities for at least 10 min (eg. heavy lifting, chopping wood, shoveling snow or digging in the garden) in the garden or yard?

☐ Days per week → Usual time spent on one of those days doing vigorous physical activities in the garden or yard ☐☐ hrs. ☐☐ min.

☐ No vigorous activity

10. During the *last 7 days*, on how many days did you do *moderate* activities (eg. carrying light loads, sweeping, washing windows, and raking in the yard) in the garden or yard?

☐ Days per week → Usual time spent on one of those days doing moderate physical activities in the garden or yard ☐☐ hrs. ☐☐ min.

☐ No moderate activity

11. During the *last 7 days*, on how many days did you do *moderate* physical activities for at least 10 min (eg. carrying light loads, washing windows, scrubbing floors and sweeping) inside your home?

☐ Days per week → Usual time spent on one of those days doing moderate physical activities inside the home ☐☐ hrs. ☐☐ min.

☐ No moderate activity inside the home

Subject ID

☐☐ ☐☐☐ ☐☐☐ ☐☐
Centre # *Community #* *Household #* *Subject #*

Subject Initials ☐☐☐
F M L

Part 4: Recreation, sport, and leisure-time physical activity

12. Not counting any walking you have already mentioned, during the *last 7 days*, on how many days did you *walk* for at least 10 minutes during in your leisure time?

☐ Days per week → Usual time spent on one of those days walking in leisure time ☐☐ hrs. ☐☐ min.

☐ No walking in leisure time

13. During the *last 7 days*, on how many days did you do *vigorous physical* activities (eg. aerobics, running, fast bicycling, or fast swimming) in your leisure time?

☐ Days per week → Usual time spent on one of those days doing vigorous physical activities in leisure time ☐☐ hrs. ☐☐ min.

☐ No vigorous activity in leisure time

14. During the *last 7 days*, on how many days did you do *moderate* physical activities for at least 10 min (eg. bicycling at a regular pace, swimming at a regular pace) in your leisure time?

☐ Days per week → Usual time spent on one of those days doing moderate physical activities in leisure time ☐☐ hrs. ☐☐ min.

☐ No moderate activity in leisure time

Part 5: Time spent sitting

15. During the *last 7 days*, how much time did you usually spend sitting on a *weekday*?

☐☐ hrs. ☐☐ min.

16. During the *last 7 days*, how much time did you usually spend sitting on a *weekend day*?

☐☐ hrs. ☐☐ min.

17. Name of Interviewer: ________ ____________________
(please print) *First Initial* *Last Name*

Interviewer code: ☐☐☐
