# Supplementary material for: Comparison of healthy lifestyle behaviors among individuals with and without cardiovascular diseases from urban and rural areas in China: A cross-sectional study
Source: PLoS One. 2017 Aug 3;12(8):e0181981. doi: 10.1371/journal.pone.0181981 (PMC5542534; doi:10.1371/journal.pone.0181981)
Supplement: S3 File — (PDF) [file pone.0181981.s005.pdf]

PURE/中国

□□	□□□	□□□	□□
中心号	*社区号*	*家庭号*	*个体号*

非常感谢您参加本项研究，您所提供的一切信息将被严格保密，并且仅当所有证明您身份的信息被删除后，才可用于研究。

个体问卷

说　明

回答问题时请在每个问题相应的方格中画“**X**”**:**

(除非另有说明**)**

☒

或者

把数字填到相应的方格中**:**

1 **8**

或者

在所给出的横线上写明答案

2007 年 4 月 19 日

个体问卷

受试者姓名缩写- F= 姓名中第一个字的首字母

M= 姓名中第二个字的首字母

L= 姓名中第三个字的首字母

3. 身份证号

如果不适用请在‘N/A’所对应方格中画‘**X**’

种族编码

01 -南亚人（印度，斯里兰卡，巴基斯坦，孟加拉国）

02- 中国人（中国，香港，台湾）

03 - 日本人

04 - 马来人

05 -亚洲其它国家（韩国，马来西亚，巴布亚新几内亚，泰国，菲律宾，印度尼西亚，尼泊尔，越南，柬埔寨，老挝，缅甸，不丹，新加坡）

06 -波斯人

07 阿拉伯人

08 -非洲黑种人

09 -非洲有色人种（仅撒哈拉非洲）

10 -欧洲人

11 -北/南美土著，或澳洲土著

12 -拉丁美洲人

13 -班图人/半班图人

14 -哈姆族人/半哈姆族人

15 -尼罗河流域人/豪撒人

16 -俾格米人

17 -斯瓦希里人

18 -其他（未列出的任何其他人种或民族）

19-汉族

20-维吾尔族

21-蒙族

22-朝鲜族

23-藏族

24-满族

25-回族

个体标识符

☐☐ ☐☐☐ ☐☐☐ ☐☐

中心号 *社区号* *家庭号* *个体号*

姓名缩写 ☐☐☐

姓 名

今天日期: ☐☐☐☐ ☐☐ ☐☐

年 月 日

1.姓名: ________________ ________________

姓 *名*

3. 身份证号（或其它有效证件）:

☐☐☐☐☐☐☐☐☐☐☐☐☐☐☐☐☐☐ **N/A** ☐

4.出生日期: ☐☐☐☐ ☐☐ ☐☐ **或** **年龄** ☐☐ 岁

年 月 日

5.性别: ☐ 女 ☐ 男

6.婚姻状况 (仅选一项):

☐ 未婚 ☐ 已婚 ☐ 同居

☐ 丧偶 ☐ 分居 ☐ 离婚

7.种族: ☐☐ ⟶ **(请参考前一页的种族编码)**

9.你完成的正规教育的程度 (仅选择最高学历) :

- ☐ 小学以下及文盲
- ☐ 小学
- ☐ 初中/高中
- ☐ 中专/技校/职高
- ☐ 大专/大学及以上
- ☐ 不详

个体问卷

11b) 职业

第 1 组: 议员，高级官员和经理

议员，高级官员

法人经理

一般经理

商人

第 2 组：专业人员

物理、数学和工程科学工作者

生命科学和卫生工作者

教学人员

其他专业人员

第 3 组：技术及相关专业人员

与物理、数学和工程科学相关专业人员/技术员

与科学工作相关人员/技术员

与生命科学和卫生专业人员/技术员

教学相关专业人员/技术员

与其他专业相关人员/技术员

第 4 组：职员

职员

顾客服务人员

第 5 组：服务工作者及商店和超市销售人员

私人的和保护性服务人员

模特，售货员和指引员

第 6 组：农业技术人员和渔业工人

面向市场的农业技术人员和渔业工人

自给自足的农业技术人员和渔业工人

第 7 组：工艺和相关贸易人员

矿石开采和建筑业的工人

金属，机械和相关贸易人员

精密、手工艺、印刷和相关贸易人员

其他工艺和相关贸易人员

第 8 组:工厂和机器操作及组装人员

固定的工厂和相关操作人员

机器操作和组装人员

驾驶员和汽车厂操作人员

第 9 组：基本（初级）职业

销售和服务基本职业

农业、渔业和相关劳动者

矿山、建筑业、制造业和交通运输业人员

第 10 组：军队

军队

第 11 组：家庭工作者

家庭主妇/家庭主男

个体标识符

中心号	社区号	家庭号	个体号	

姓名缩写 姓 名

11b) 请指出对您所从事主要职业的最恰当描述

(请参考上一页对组别的定义以及操作手册中的详细定义)

☐ 第 1 组 ☐ 第 2 组 ☐ 第 3 组 ☐ 第 4 组 ☐ 第 5 组

☐ 第 6 组 ☐ 第 7 组 ☐ 第 8 组 ☐ 第 9 组 ☐ 第 10 组 ☐ 第 11 组

如果您的职业属于第 11 组 (家庭工作者)，请跳到 13

12. 您现在是否有工作?

☐ 否 → **(回答 12a - 12b)** ☐ 是 → **跳到 13**

a) 您是否退休或者由于年龄过大而停止工作? ☐ 否 ☐ 是

b) 您是否由于患病而停止工作? ☐ 否 ☐ 是

个体标识符 □□ □□□ □□□ □□ 姓名缩写 □□□

中心号 社区号 家庭号 个体号 *姓 名*

13. 目前的行为能力障碍(有障碍者填是，没有障碍者填否)：

	否	是
**a)** 用手指抓东西或搬东西是否存在问题?	□	□
**b)** 散步是否有困难?	□	□
**c)** 弯腰从地板上捡东西是否有困难?	□	□
**d)** 是否要借助拐仗等物品才能行走?	□	□
**e)** 戴眼镜是否能阅读或看盘子中的单个米粒或谷粒?	□	□
**f)** 在屋里戴着眼镜看对面的人有困难?（3.5 米远）	□	□
**g)** 是否存在交流和理解上的障碍?	□	□
**h)** 在正常谈话中能听清对方所说的话吗?	□	□

既往史(个人病史)

14.您最近六个月是否有以下症状?

	否	是		否	是
**a)** 常规活动时胸痛或胸闷	□	□	**i)** 呕吐	□	□
如果是,——▶疼痛是否扩散到后背、颈部或上臂内侧	□	□	**j)** 无食欲	□	□
**b)** 常规活动时感到气短	□	□	**k)** 牙疼或牙龈出血	□	□
**c)** 咳嗽至少 2 周	□	□	**l)** 黄疸	□	□
**d)** 咳嗽带痰	□	□	**m)** 排尿时有烧灼感	□	□
**e)** 痰里有血丝	□	□	**n)** 脚肿	□	□
**f)** 呼吸困难或哮鸣音	□	□	**o)** 脸肿	□	□
**g)** 早晨咳嗽伴胸闷	□	□	**p)** 尿中有血	□	□
**h)** 腹泄三天以上	□	□	**q)** 最近一年非自愿的体重下降大于 3 公斤	□	□

15. 在最近的 2 年中是否每年都有连续 3 个月的时间出现咳痰? 否 □ 是 □

16a) 目前您戴眼镜或隐型眼镜吗? 否 □ 是 □

b) 是否使用助听器? 否 □ 是 □

个体问卷

癌症部位

1= 口腔
2= 食道
3= 胃
4= 小肠
5= 大肠包括直肠
6= 胰腺
7= 肝脏
8= 肺
9= 乳房
10= 子宫颈/子宫/卵巢
11= 前列腺
12= 头、颈
13= 其他，请注明

个体标识符 ☐☐ ☐☐☐ ☐☐☐ ☐☐ 姓名缩写 ☐☐☐

中心号 社区号 家庭号 个体号 *姓 名*

17. 您是否曾被诊断患有下列疾病？(填写所有符合的项目)

(不到一年者，填"0")

	否	是	从诊断至今几年了		否	是	从诊断至今几年了
a)糖尿病	☐	☐	☐☐	i) 慢性阻塞性肺病	☐	☐	☐☐
b)高血压/血压高	☐	☐	☐☐	j) 哮喘	☐	☐	☐☐
c)脑卒中	☐	☐	☐☐	k) 肺结核	☐	☐	☐☐
d)心绞痛/心脏病发作/冠心病	☐	☐	☐☐	l) 疟疾	☐	☐	☐☐
e)心衰	☐	☐	☐☐	m)南美锥虫病	☐	☐	☐☐
f) 其它心血管疾病	☐	☐	☐☐	n) HIV/艾滋病 未回答 ☐	☐	☐	☐☐
h)肝炎/黄疸	☐	☐	☐☐				
g)癌症	☐	☐	☐☐				

请参考上一页对癌症部位的定义 ☐☐ *部位* ________ *其他，请注明*

18. 在最近的一个月里是否有规律(至少一周一次)的服用某种药物？ ☐否 → **跳到 19** ☐是

a) 如果回答是，请指明用药种类:

	否	是
降压药	☐	☐
降脂药	☐	☐
治疗中风药	☐	☐
降糖药	☐	☐
治疗哮喘药	☐	☐
中药	☐	☐
其他药物	☐	☐ → 如回答是，请注明 ____________________
未 知 药	☐	☐

个体问卷

18b) 如果所使用的药物名称未知，在列表中请注明未知.

个体标识符 □□ □□□ □□□ □□

中心号　社区号　家庭号　个体号

姓名缩写 □□□

姓　名

18b) 请列出最近一个月中，您每周至少服用一次的所有药物

i) ____________ **ii)** ____________

iii) ____________ **iv)** ____________

v) ____________ **vi)** ____________

vii) ____________ **viii)** ____________

男性请直接回答问题 23

只适用于女性 (问题 19 - 22)

19. 您现在是否怀孕? □ 否 □ 是 → **跳到 21**

20. 您是否还有月经? □ 否 → **(回答 20a)** □ 是 → **跳到 21**

a)您绝经已经几年了? □□ 年 **(不到 1 年填'0'，大于 1 年半填'2'，以此类推)**

21. 您是否使用过口服或注射避孕药? □ 否 □ 是

(不考虑使用时间长短)

22a)您生育过多少个成活的孩子? □□ 男孩 □□ 女孩

(仅包括存活大于 1 个月的)

b) 您是否用母乳喂养过您的孩子? □ 否 □ 是

23. 您骑摩托车/机动脚踏两用车时是否戴头盔?

a) 作为驾驶员 □ 否 □ 是 □ 不适用

b）作为乘客 □ 否 □ 是 □ 不适用

24. 您开轿车/吉普车时是否系安全带?

a) 作为驾驶员 □ 否 □ 是 □ 不适用

b) 作为乘客 □ 否 □ 是 □ 不适用

个体问卷

25. 意外事故和伤害

受伤地点

1= 工厂/工业场所
2= 办公室
3= 农田/农场
4= 家中
5= 公路
6= 运动/比赛 例如：跑道，球场，运动场，等等.
7= 公共建筑
8= 矿山/采石场
9= 建筑场所 例如：建筑工地,筑路工地，等等.
10 = 其他

受伤类型

1= 烧伤
2= 烫伤
3= 骨折
4= 肌肉和韧带扭伤/撕裂
5= 刀伤和刺伤
6= 撞伤和擦伤
7= 窒息
8= 头部损伤 (但没有失去意识/知觉)
9= 头部损伤 (在一段时间内失去意识/知觉)

个体标识符

中心号	社区号	家庭号	个体号

姓名缩写 姓 名

25. 在过去 12 个月中，您是否受过致使您正常活动受到限制的严重伤害?

(填写所有符合的项目) ☐ 否 → 跳到 26 ☐ 是 → (回答 25a - 25s)

如果回答是，请提供详细信息: 请参考上一页对受伤地点和类型的定义

受伤原因		地点	类型	不能正常工作或活动的时间（天数）
a) 机动车事故（作为乘员）	☐ 否 ☐ 是 →			
b) 机动车事故（作为行人）	☐ 否 ☐ 是 →			
c) 被外物击打	☐ 否 ☐ 是 →			
d) 爆炸	☐ 否 ☐ 是 →			
e) 自然/环境因素 大风/龙卷风/闪电，等等.)	☐ 否 ☐ 是 →			
f) 窒息	☐ 否 ☐ 是 →			
g) 中毒	☐ 否 ☐ 是 →			
h) 蛇/蝎子咬伤	☐ 否 ☐ 是 →			
i) 坠落伤	☐ 否 ☐ 是 →			
i) 火/火灾，烟熏	☐ 否 ☐ 是 →			
k) 身体遭袭击（枪击，诱拐，等等.)/暴力犯罪	☐ 否 ☐ 是 →			
l) 家庭暴力（被家庭成员殴打）	☐ 否 ☐ 是 →			
m)溺水/被水淹	☐ 否 ☐ 是 →			
n) 烫或腐蚀性液体/食物/物品	☐ 否 ☐ 是 →			
o) 压伤（大石头，建筑材料，等等.）	☐ 否 ☐ 是 →			
p) 由机器导致的意外伤害	☐ 否 ☐ 是 →			
q) 试图自杀	☐ 否 ☐ 是 →			
r) 武装冲突	☐ 否 ☐ 是 →			
s) 其他(请标明) ________	☐ 否 ☐ 是 →			

个体问卷

骨折的部位

1= 臀/骨盆

2= 大腿（股）

3= 腿（胫）

4= 前臂

5= 手腕

6= 手/手指

7= 椎骨 (背部)

8= 其他

骨折: 如果调查对象由于摔倒受伤，无法判断是韧带撕裂或是骨折时，只有当医生证实为骨骼受伤时，才能认为是骨折。

27c) 规律吸烟: 指每天至少吸一支及以上烟.

如何填写持续时间:

对于那些吸烟时间不到 1 年的被调查者, 持续时间请填写 “0”

个体标识符

□□ □□□ □□□ □□
中心号　社区号　家庭号　个体号

姓名缩写 □□□
姓　名

26.您是否曾经骨折过? □ 否 (跳到 27) □ 是 (如果是，回答 a)、b)和 c)

a) 骨折次数 □□

b) 上次骨折距现在多少年 □□ (年)

c) 请您列出距现在最近的一次骨折中您骨折的部位（如果多于 3 处，请按严重程度列出前 3 个） (部位) 若是其他，请注明

□ → ________________

□ → ________________

□ → ________________

请参考上一页对骨折部位的定义

吸烟

27. 哪一项是对您的吸烟史最合适的描述?

a) □ 以前吸烟　□ 现在吸烟　□ **从不吸烟** → **跳到 28**

b) 从几岁开始吸烟? □□ 岁

c) 您经常吸下列哪类型的烟? (填写所有符合的项目)

	平均每天数量	持续时间 (年)	何时停止 (几年前)	如果小于 1 年 (几个月之前)
		只有已经停止吸烟者填写		
(i) 香烟 (各种类型)	□□□ 支	□□	□□	□□
(iii) 雪茄	□□□ 支	□□	□□	□□
(iv) 烟斗	□□□ 支	□□	□□	□□
(v) 水烟袋	□□□ 次数	□□	□□	□□
(vi) 卷烟叶	□□□ 支	□□	□□	□□
(ix)其他____ *请注明*	□□□	□□	□□	□□

1 克烟叶(去梗)=1 支香烟

个体标识符

□□ □□□ □□□ □□

中心号 社区号 家庭号 个体号

姓名缩写 □□□

姓 名

从未吸烟或过去吸烟者回答问题 28

28.在过去 12 个月中，您是否经常(至少每周一次) 处于被动吸烟的状态？

(“被动吸烟” 定义为持续吸入别人所吸的烟，时间在 5 分钟以上.)

□否 ⟶ **跳到 29** □ 是 ⟶请回答问题 **28a**

a) 在过去 12 个月中，您处于被动吸烟的时间？

(“被动吸烟” 定义为持续吸入别人所吸的烟，时间在 5 分钟以上.)

只选择一项

□每周 1-2 次 □每周 3-6 次 □每天至少 1 次 □每天 2-3 次 □每天多于 4 次

29. 不适用于中国

个体问卷

30c) 饮酒: 每月至少喝一次酒被定义为经常饮酒**.**

个体标识符

中心号 □□ 社区号 □□□ 家庭号 □□□ 个体号 □□

姓名缩写 □□□ 姓 名

30. 哪一项是对您的饮酒史最合适的描述？

a) □ 以前饮酒 □ 现在饮酒 □ 从不饮酒 ⟶ **跳到 31**

b) 从几岁开始饮酒？ □□ 岁

c) 您经常饮用下列哪种类型的酒？ (填写所有符合的项目)

酒的类型	每杯大概量	饮用频率(只选一项) 每天	每周	每月	每次平均几杯	持续时间(年)	只有已经停止饮酒者填写 何时停止 (几年前)
(ii) 葡萄酒	125ml	□	□	□	□□	□□	□□
(iii) 白酒 ≤ 38 度	125ml	□	□	□	□□	□□	□□
(iv) 白酒 > 38 度	125ml	□	□	□	□□	□□	□□
(vi) 啤酒	375ml	□	□	□	□□	□□	□□

d) 您是否每个月至少有 1 次一天饮酒多于 5 杯？ □ 否 ⟶ 跳到 31 □ 是

↓ 若是,(i,ii)

i) 一个月中您有多少天时间是每天饮酒多于 5 杯？ □□

ii) 您每次平均饮酒多少杯？ □□

31 a) 在您最长时间睡觉或夜间睡觉的时候，您通常几点钟上床？ □□:□□ (00:00-23:59)

b) 在您最长时间睡觉或夜间睡觉的时候，您通常几点钟起床？ □□:□□ (00:00-23:59)

c) 您是否经常打盹或午睡？ □ 否 □ 是 *总的打盹时间* ⟶ □□□ 分钟

35. <u>**市民组织:**</u>　非营利性的，自愿发起，实行自助的组织或俱乐部.

<u>**宗教组织:**</u>　以宗教信仰为基础的不同类型的正式或非正式的组织.

个体标识符

☐☐	☐☐☐	☐☐☐	☐☐
中心号	*社区号*	*家庭号*	*个体号*

姓名缩写 ☐☐☐ *姓 名*

33. 请回答以下问题:(每问只选一个答案)

	非常不同意	有些不同意	有些同意	非常同意
(i) 人们通常都是诚实的、并且愿意帮助他人.	☐	☐	☐	☐
(ii) 如果我为别人做了好事, 我会期待他们尊敬我, 并且像我对待他们一样来对待我.	☐	☐	☐	☐

**34a) 电视, 广播, 报纸或杂志上的广告，帮我决定购买下列物品：**(每问只选一个答案)					不适用
(i) 烹饪用油	☐	☐	☐	☐	☐
(ii) 面粉	☐	☐	☐	☐	☐
(iii) 大米	☐	☐	☐	☐	☐
**b) 通过电视, 广播, 报纸或杂志上的广告，影响我购买下列物品：**(每问只选一个答案)					
(i) 软饮料	☐	☐	☐	☐	☐
(ii) 点心/零食	☐	☐	☐	☐	☐
(iii) 香烟	☐	☐	☐	☐	☐
(iv) 酒	☐	☐	☐	☐	☐

35. 在有困难时, 您会依靠哪里提供的帮助? (参考上一页的定义)

(i) 市民组织: 请注明________________

☐无 ☐一点 ☐ 中等的 ☐ 大量的

(ii) 宗教组织: 请注明_________________

☐无 ☐一点 ☐ 中等的 ☐ 大量的

个体标识符

□□	□□□	□□□	□□
中心号	*社区号*	*家庭号*	*个体号*

姓名缩写 □□□
姓　　名

36. 您在最近的 12 个月中是否有如下经历?

	未回答	否	是	
(i) 失去工作	□	□	□	
(ii) 退休	□	□	□	
(iii) 产量减少或生意失败	□	□	□	
(iv) 遭到入室抢劫	□	□	□	
(v) 分居或离婚	□	□	□	
(vi) 其他主要家庭内部冲突	□	□	□	→ 请具体注明________
(vii) 主要家庭成员受伤或患病	□	□	□	
(viii) 遭暴力伤害	□	□	□	
(ix) 武装冲突或战争	□	□	□	
(x) 丧偶	□	□	□	
(xi) 亲密的家庭成员的死亡或大病	□	□	□	
(xii) 其他主要压力	□	□	□	→ 请具体注明________
(xiii) 家庭成员结婚	□	□	□	
(xiv) 获得新工作	□	□	□	
(xv) 出生新家庭成员	□	□	□	
(xvi) 与家庭分离	□	□	□	
(xvii) 食物不安全	□	□	□	

个体标识符

中心号	社区号	家庭号	个体号	姓名缩写（姓 名）

37. 请回答下列问题: (每问只选一个答案)

在回答下列问题时，压力感被定义为感觉易怒或者充满焦虑，或在工作或家里出现睡眠困难.

	未回答	从来没有过	有时有	周期性的	一直有
**a)** 最近 12 个月中您在工作时是否经常感到有压力? (如果不适用请在这里作标记: 例如，不再工作) ☐	☐	☐	☐	☐	☐
**b)** 最近 12 个月中您在家中时是否经常感到有压力?	☐	☐	☐	☐	☐

38. 最近 12 个月中您承受经济压力有多大?

☐ 不回答 ☐ 很少或没有 ☐ 中度 ☐ 高度或严重

39. 在过去的 12 个月中，您是否连续两周或者在更长时间里感到悲哀、忧郁，或消沉?

☐ 否 ☐ 是 ⟶ 如果回答是，在这段时间里，您是否:

	未回答	否	是
**a)** 对大多数曾给您带来快乐的事情失去兴趣? 如业余爱好、工作或活动	☐	☐	☐
**b** 感到劳累或无精打采?	☐	☐	☐
**c)** 体重增加或减少?	☐	☐	☐
**d)** 感觉比平时入睡困难?	☐	☐	☐
**e)** 不能向往常那样集中精神?	☐	☐	☐
**f)** 经常考虑死(自己，他人或者通常意义上的死亡)	☐	☐	☐
**g)** 有失落感，觉得自己不好或没有价值?	☐	☐	☐

个体标识符

中心号	*社区号*	*家庭号*	*个体号*

姓名缩写 *姓 名*

40. 请回答下列问题: (每问只选一个答案)

	非常不同意	有些不同意	有些同意	非常同意
**a)** 在离家步行容易达到的（15 分钟以内）商店，能够购买绝大多数的日常用品（食物，生活必需品等）.	☐	☐	☐	☐
**b)** 由于速度和/或交通量问题，在我所居住的环境里步行或骑车非常困难.	☐	☐	☐	☐
**c)** 通常我的居住环境远离污染（垃圾、空气或者噪音污染）.	☐	☐	☐	☐
**d)** 我所居住的街道，在晚上非常明亮.	☐	☐	☐	☐
**e)** 在我所居住的环境里散步时可以看到其他人.	☐	☐	☐	☐
**f)** 在我所居住的环境里散步时可以和其他人聊天.	☐	☐	☐	☐
**g)** 在我所居住的环境里犯罪率很高.	☐	☐	☐	☐
**h)** 无人看管的狗在我所居住的环境里是一个问题.	☐	☐	☐	☐

42b) 健康史:

癌症部位

1= 口腔
2= 食道
3= 胃
4= 小肠
5= 大肠包括直肠
6= 胰腺
7= 肝脏
8= 肺
9= 乳房
10= 子宫颈/子宫/卵巢
11= 前列腺
12= 头、颈
13= 其他，请注明

个体标识符

中心号	社区号	家庭号	个体号
☐☐	☐☐☐	☐☐☐	☐☐

姓名缩写 ☐☐☐ *姓 名*

41. 如果步行，从您家到最近的下列地点要花多长时间?

	分钟	不知道		分钟	不知道
i)杂货店/便利店/超市	☐☐☐	☐	iv) 音像商店	☐☐☐	☐
ii) 银行	☐☐☐	☐	v) 非快餐店	☐☐☐	☐
iii) 邮局	☐☐☐	☐	vi) 快餐店	☐☐☐	☐

42a) 兄弟姐妹总数(包括本人) ☐☐

b) 家族史: 请完成所有关于双亲、兄弟姐妹的问题，无论活着或死亡

	父亲			母亲			兄弟姐妹			如果是 → 兄弟姐妹中有多少人是此种情况
	不知道	否	是	不知道	否	是	不知道	否	是	
糖尿病	☐	☐	☐	☐	☐	☐	☐	☐	☐	☐☐
冠心病	☐	☐	☐	☐	☐	☐	☐	☐	☐	☐☐
高血压	☐	☐	☐	☐	☐	☐	☐	☐	☐	☐☐
脑卒中	☐	☐	☐	☐	☐	☐	☐	☐	☐	☐☐
癌症 请参考上一页对癌症部位的定义	☐	☐	☐	☐	☐	☐	☐	☐	☐	☐☐
→ 如果是，注明部位 (如兄弟姐妹中有多个癌症，只写一个)	☐☐			☐☐			☐☐			
	↓ ______ 其他,请注明			↓ ______ 其他，请注明			↓ ______ 其他，请注明			

个体问卷

如果被调查者拒绝进行某项测量，在相应问题的每个空格中都填“0”

更多的详细说明请参考操作手册

个体标识符 ☐☐ ☐☐☐ ☐☐☐ ☐☐ 姓名缩写 ☐☐☐

中心号 *社区号* *家庭号* *个体号* *姓 名*

43. 体格检查

a) 坐位右臂血压
#1 ☐☐☐ ☐☐☐ 毫米汞柱
收缩压 *舒张压*
#2 ☐☐☐ ☐☐☐ 毫米汞柱
收缩压 *舒张压*

b) 心率
#1 ☐☐☐ 次/分钟
#2 ☐☐☐ 次/分钟

c) 腰围
#1 ☐☐☐.☐ 厘米
#2 ☐☐☐.☐ 厘米
→ ☐ 少量或未穿 ☐ 全部衣物

d) 体重 ☐☐☐.☐ 公斤 → ☐ 少量或未穿 ☐ 全部衣物

e)臀围
#1 ☐☐☐.☐ 厘米
#2 ☐☐☐.☐ 厘米
→ ☐ 少量或未穿 ☐ 全部衣物

f) 身高 ☐☐☐.☐ 厘米 (脱鞋)

44. a) 右上臂围 ☐☐.☐ 厘米 b) 右小腿围 ☐☐.☐ 厘米

c) 头围: ☐☐☐.☐ 厘米

个体标识符 ☐☐ ☐☐☐ ☐☐☐ ☐☐ 姓名缩写 ☐☐☐

中心号 *社区号* *家庭号* *个体号* *姓* *名*

47. 握力 (最大收缩): <u>请标明哪只手为优势手</u>

a) 非优势手:

#1 ☐☐ 公斤

#2 ☐☐ 公斤

#3 ☐☐ 公斤

b) 优势手:

#1 ☐☐ 公斤

#2 ☐☐ 公斤

#3 ☐☐ 公斤

个体问卷

如果被调查者拒绝进行某项测量, 在相应问题的每个空格中都填“0”

更多的详细说明请参考说明手册

48. 肺功能测定:

美国胸科协会提供的肺功能合格标准:
只有在不出现下列情况时的结果才认为是可接受的:

1. 呼气时咳嗽
2. 早期终止或中断
3. 没有持续用力
4. 漏气
5. 口器被阻塞

肺功能测定注意事项 ：

1、 室内温度不能过高

2、 随访季节应与入选时尽量一致

3、 装有满口假牙者或前部假牙者，应将假牙拿出

4、 严重的肺气肿、感冒、发烧、慢性支气管炎急性发作者不作

5、 严重高血压者酌情慎作

6、 一定要避免吹气时，受试者发生气胸

个体标识符

☐☐ ☐☐☐ ☐☐☐ ☐☐ 姓名缩写 ☐☐☐

中心号 社区号 家庭号 个体号 *姓 名*

48. 肺功能测定:

a) FEV1 (升): #1☐. ☐☐ #2 ☐. ☐☐ #3 ☐. ☐☐

b) FEV1 是否符合 ATS 标准?

☐ 否 →(回答问题(i)至(iii) ☐ 是 →跳到c)

不符合 ATS 标准的原因: (填写所有符合项目)

i) 咳嗽 ☐

ii) 各次测量值之差大于 0.2L ☐

iii) 少于 3 个测量值 ☐

c) FVC (升): #1☐. ☐☐ #2 ☐. ☐☐ #3 ☐. ☐☐

d) FVC 是否符合 ATS 标准?

☐ 否 →(回答(i)至(iii) ☐ 是 →跳到 e)

不符合 ATS 标准的原因: (填写所有符合项目)

i) 咳嗽 ☐

iii) 少于 3 个测量值 ☐

ii) 各次测量值之差大于 0.2L ☐

e) PEFR (升/分钟): #1 ☐☐☐ #2 ☐☐☐ #3 ☐☐☐

f) PEFR 是否符合 ATS 标准?

☐ 否 →(回答(i)至(ii) ☐ 是 →回答问题 49

不符合 ATS 标准的原因: (填写所有符合项目)

i) 咳嗽 ☐

ii) 少于 3 个测量值 ☐

个体标识符 ☐☐ ☐☐☐ ☐☐☐ ☐☐

中心号 *社区号* *家庭号* *个体号*

姓名缩写 ☐☐☐

姓 名

50 是否做了心电图(ECG)? 否 ☐ → **跳到 51** 是 ☐

a) 2 0 ☐☐ ☐☐ ☐☐ →
年 月 日

ECG:File 标签贴于此

b) 请抄写 ECG 标签号码: ☐☐☐☐☐☐

51a) 是否获得了血样? 否 ☐ → **跳到 52** 是 ☐

b) ☐ **空腹样本** ☐ **非空腹样本**

c) 2 0 ☐☐ ☐☐ ☐☐ **时间** ☐☐:☐☐ → **从上次进食/喝饮料(不包括水)到现在的小时数** ☐☐
年 月 日 **(00:00-23:59)**

d) 请抄写血样标签号码: ☐☐☐☐☐☐

血样标签 贴于此

52 a) 是否获得了尿样(中段尿)? 否 ☐ → **跳到 53** 是 ☐

b) ☐ **空腹样本** ☐ **非空腹样本**

c) 请抄写尿样标签号码: ☐☐☐☐☐☐

尿样标签 贴于此

53. 调查员姓名: ________ ________
姓 名

调查员编码: ☐☐☐

PURE/中国

非常感谢您参加本项研究，您所提供的一切信息将被严格保密，并且仅当所有证明您身份的信息被删除后，才可用于研究。

体力活动问卷

说　明

回答问题时请在每个问题相应的方格中画 **X:**

(除非另有说明**)**

☒

或者

把数字填到相应的方格中**:**

[1][8]

或者

在所给出的横线上写明答案

2005 年 2 月 2 日

体力活动问卷

受试者姓名缩写- F=姓名中第一个字的首字母

M=姓名中第二个字的首字母

L= 姓名中最后一个字的首字母

个体标识符

☐☐ 中心号　☐☐☐ 社区号　☐☐☐ 家庭号　☐☐ 个体号

姓名缩写 ☐☐☐ *姓　名*

今日日期: ☐☐☐☐ *年* ☐☐ *月* ☐☐ *日*

1. 在您的工作生涯中，您最主要的职业或工作是什么?

____

第 1 部分: 与工作相关的体力活动

(在家庭外从事的无论有无薪水报酬) *家庭劳动除外*

2. 您现在是否有固定工作，或者是否在家庭以外从事无薪水报酬的工作?

☐ 否 → **跳到第 2 部分**　　☐ 是 → **继续回答问题 3**

3. 在过去的 7 天里，作为您工作的一部分，共有几天从事至少持续 10 分钟重体力活动?（例如举重物、挖掘、工作量大的建筑工作）

在上述问题所述的时间里，通常您每天的工作中有多长时间在从事重体力活动

☐ 每周的天数 → ☐☐ 小时 ☐☐ 分钟

☐ 工作中无重体力活动

4. 在过去的7天里，作为您工作一部分共有几天从事至少持续10分钟中等体力活动?（例如搬运轻物品）*请不要把步行包括在内*

在上述问题所述的时间里，通常您每天的工作中有多长时间在从事中等体力活动

☐ 每周的天数 → ☐☐ 小时 ☐☐ 分钟

☐ 工作中无中等体力活动

5. 在过去的 7 天里，作为您工作一部分，共有几天每日步行至少持续 10 分钟?

请不要将上下班过程中的步行时间计算在内

在上述问题所述的时间里，通常您每天的工作中有多长时间在步行

☐ 每周的天数 → ☐☐ 小时 ☐☐ 分钟

☐ 工作中不需要步行

个体标识符

☐☐ ☐☐☐ ☐☐☐ ☐☐ 姓名缩写 ☐☐☐

中心号 *社区号* *家庭号* *个体号* *姓 名*

第 2 部分：与交通工具相关的体力活动

6. 在过去的 7 天里，您有几天乘坐机动车去工作、购物、看电影等等？

在上述问题所述的时间里，您每天通常有多长时间以机动车作为交通工具

☐ 每周的天数 ——→ ☐☐ 小时 ☐☐ 分钟

☐ 未用机动车作为交通工具

7. 在过去的 7 天里，您共有几天是一次性骑自行车超过 10 分钟从某地到某地？

在上述问题所述的时间里，您每天通常有多长时间以自行车作为交通工具

☐ 每周的天数 ——→ ☐☐ 小时 ☐☐ 分钟

☐ 未用自行车作为交通工具

8. 在过去的 7 天里，您共有几天是一次性步行超过 10 分钟从某地到某地？

在上述问题所述的时间里，您每天通常有多长时间在外出时采用步行

☐ 每周的天数 ——→ ☐☐ 小时 ☐☐ 分钟

☐ 外出从未采用步行

个体标识符

□□	□□□	□□□	□□	姓名缩写	□□□
中心号	*社区号*	*家庭号*	*个体号*		*姓　名*

第 3 部分：家务劳动、房屋保养和为家庭的付出

9. 在过去的 7 天里，您共有几天在花园或院子中从事 10 分钟以上的剧烈的体力活动？（例如举重物、砍伐树木、铲雪或者在花园中挖地）

在上述问题所述的时间里，您每天通常有多长时间在花园或院子中从事剧烈的体力活动

☐ 每周的天数 ———————→ □□ 小时 □□ 分钟

☐ 无剧烈的体力活动

10. 在过去的 7 天里，您共有几天在花园或院子中从事 10 分钟以上的中等的体力活动？（例如搬运轻物品、打扫、清洗窗户、和在院子里耙草）

在上述问题所述的时间里，您每天通常有多长时间在花园或院子中从事中等体力活动

☐ 每周的天数 ———————→ □□ 小时 □□ 分钟

☐ 无中等的体力活动

11. 在过去的 7 天里，您共有几天在您的屋子里面从事 10 分钟以上的中等的体力活动？（例如搬运轻物品、清洗窗户、擦地和打扫）

在上述问题所述的时间里，您每天通常有多长时间在家里从事中等体力活动

☐ 每周的天数 ———————→ □□ 小时 □□ 分钟

☐ 无在家中的中等的体力活动

个体标识符

□□ 中心号　□□□ 社区号　□□□ 家庭号　□□ 个体号

姓名缩写 □□□ 姓 名

第 4 部分：娱乐、运动和空闲时间的体力活动

12. 不算以上提到的所有步行时间，在过去的 7 天里，在空闲时间里，您共有几天每日步行超过 10 分钟？

在上述问题所述的时间里，您每天通常有多长时间在空闲时步行

□ 每周的天数 → □□ 小时 □□ 分钟

□ 在空闲时间没有步行

13. 在过去的 7 天里，您共有几天在空闲时间里进行剧烈体力活动？（例如有氧运动、跑步、快速骑车、或者快速游泳）

在上述问题所述的时间里，您每天通常有多长时间在空闲时进行剧烈体力活动

□ 每周的天数 → □□ 小时 □□ 分钟

□ 在空闲时间无剧烈的体力活动

14. 在过去的 7 天里，您共有几天在空闲时间中进行至少 10 分钟一次的中等体力活动？（例如以通常速度骑车、以通常速度游泳）

在上述问题所述的时间里，您每天通常有多长时间在空闲时进行中等的体力活动

□ 每周的天数 → □□ 小时 □□ 分钟

□ 在空闲时间无中等体力活动

第 5 部分：处于坐姿的时间

15. 在过去的 7 天里，您每个工作日有多长时间处于坐姿？

□□ 小时 □□ 分钟

16. 在过去的 7 天里，您每个休息日有多长时间处于坐姿？

□□ 小时 □□ 分钟

17.调查员姓名： ________ ________ 　　**调查员编码：** □□□

姓　名
